# Cytosolic phospholipase A2 as a therapeutic target for degenerative joint diseases

**DOI:** 10.1038/s41413-025-00470-9

**Published:** 2025-10-15

**Authors:** Guiwu Huang, Chaopeng He, Wenyu Fu, Jingwei Bi, Jianji Wang, Daniel H. Wiznia, Chuan-ju Liu

**Affiliations:** 1 Department of Orthopaedics and Rehabilitation, Yale University School of Medicine, New Haven, CT USA; 2 Department of Joint Surgery, The First Affiliated Hospital of Sun Yat-sen University, Sun Yat-sen University, Guangzhou, China; 3 Department of Orthopedics, The Second Xiangya Hospital of Central South University, Changsha, China

**Keywords:** Homeostasis, Metabolic syndrome

## Abstract

Osteoarthritis (OA) and intervertebral disc degeneration (IVDD) are degenerative musculoskeletal disorders characterized by degeneration of cartilaginous tissues and inflammation. While inflammation is implicated in the pathogenesis of OA and IVDD, and cytosolic phospholipase A2 (cPLA2) is a key mediator of inflammation, direct evidence linking cPLA2 to chondrocyte homeostasis and cartilage degeneration is lacking. This study aims to investigate the role of cPLA2 in chondrocytes and its contribution to the development of cartilage degenerative conditions such as OA and IVDD. Here, single-cell RNA sequencing was used to examine cPLA2 expression in chondrocytes. To explore its importance in chondrocytes and OA/IVDD, various cell-based assays and genetically modified mouse models with age-related and surgically induced OA/IVDD were employed. Furthermore, the therapeutic potential of fexofenadine, an over-the-counter drug recently identified as a cPLA2 inhibitor, was explored in these models. cPLA2 is predominantly expressed in prehypertrophic chondrocytes, characterized by elevated levels of cartilage degeneration markers and senescence-related genes. Genetic deletion and pharmacological inhibition of cPLA2 reduced inflammation induced catabolic activity and senescence in chondrocytes, as well as cartilage degeneration in various OA and IVDD models. This study identifies cPLA2 as a pivotal driver of cartilage degeneration and senescence in OA and IVDD, highlighting its potential as a dual-action therapeutic target that suppresses both inflammation and senescence to preserve cartilage integrity. These findings position cPLA2 as a promising candidate for developing disease-modifying therapies for cartilage degenerative conditions such as OA and IVDD.

## Introduction

Osteoarthritis (OA) is a chronic, progressively degenerative disease affecting the entire joint, representing a leading cause of disability among the global elderly and placing a substantial burden on healthcare systems.^1–4^ Degenerative changes in joint tissues result from the complex interplay of inflammatory responses, mechanical stress, and metabolic abnormalities.^5–7^ This interplay is often compounded by a mismatch between the body’s adaptive capacity and rapidly evolving environmental conditions.^8–10^ Pathologically, OA is characterized by the degeneration of cartilage and inflammation of the surrounding tissues, leading to clinical symptoms including swelling, synovitis and inflammatory pain.^11^ Despite various efforts, including cartilage transplantation and stem cell therapy, cartilage repair and delaying the progression of OA remain significant challenges.^12–14^ Current therapies, such as nonsteroidal anti-inflammatory drugs (NSAIDs) and analgesics, primarily target symptom relief, without preventing further cartilage or joint damage.^15,16^ The lack of disease-modifying OA drugs underscores the urgent need for new therapeutic targets and strategies that can alter the disease trajectory and improve the long-term outcome of OA patients.^17–19^

Phospholipase A2 (PLA2), a key enzyme involved in generating a wide range of inflammatory mediators, plays an important role in the pathophysiology of both rheumatoid arthritis (RA) and OA.^20^ The PLA2 family includes three cytosolic isoforms and ten secretory isoforms: IB, IIA, IIC, IID, IIE, IIF, III, V, X, and XII.^21^ Secretory phospholipase A2 (sPLA2) has been previously reported to play a regulatory role in the progression of OA, and inhibition of sPLA2 activity holds potential for blocking the production of inflammatory mediators.^22–24^ And cytosolic phospholipase A2 (cPLA2), a member of type IV phospholipase A2 family, plays a pivotal role in inflammatory responses^25–27^ due to its unique specificity for arachidonic acid (AA) production, which is released from membrane phospholipids.^28,29^ AA serves as a precursor for lipid mediators such as prostaglandins (PGE2), leukotrienes (LTB4), and thromboxane, which participate in inflammatory response by regulating pro or anti-inflammatory effects.^7,30–33^ Additionally, AA has been shown to regulate immune cell activation and synovium inflammation in RA patients.^34^

We recently reported that pharmacologically targeting inflammatory TNFα/cPLA2 signaling pathway using the FDA-approved over-the counter drug fexofenadine (FFD) holds promise as a therapeutic strategy in mouse models of inflammatory arthritis and inflammatory bowel disease.^25,35^ Specifically, FFD inhibited TNFα-stimulated inflammatory response in macrophages through directly binding to cPLA2, thereby suppressing cPLA2 activity and reducing AA production. Additionally, TNFα/cPLA2 signaling pathway is believed to contribute to OA pathogenesis. However, direct evidence linking cPLA2 to OA pathogenesis, especially its regulatory effects on chondrocytes, has not been reported, and the potential of targeting cPLA2 as a therapeutic target for OA remains to be explored.

Both OA and intervertebral disc degeneration (IVDD) are degenerative musculoskeletal disorders characterized by degeneration of cartilaginous tissues, inflammation, and extracellular matrix breakdown, leading to pain and functional impairment.^36^ Leveraging both OA and IVDD mouse models allow us to evaluate therapeutic strategies targeting shared molecular pathways, such as inflammatory signaling and extracellular matrix degradation, potentially leading to the development of treatments that address multiple aspects of joint degeneration.

In this study, cPLA2 is identified as a critical mediator of senescence responses and cartilage degeneration in human OA cartilage through an integrative analysis of single-cell RNA sequencing (scRNA-seq) data from healthy and OA cartilage. Our findings reveal that cPLA2 regulates expression of genes associated with chondrocyte metabolism and senescence. Furthermore, genetic deletion or pharmacological inhibition of cPLA2 significantly mitigates structural joint damage, disc degeneration, and pain in both surgically-induced and age-related murine OA and IVDD models. Collectively, our results establish cPLA2 as a previous unrecognized gene associated with both OA and IVDD, highlighting its pivotal role in regulating chondrocyte biology. These finding underpin the therapeutic potential of targeting cPLA2 for treating degenerative joint disorders, offering new avenues for intervention in conditions marked by cartilage degeneration and inflammation.

## Results

### cPLA2 is predominantly expressed in prehypertrophic chondrocytes and associated with cartilage degeneration and senescence responses

**Fig. 1:**
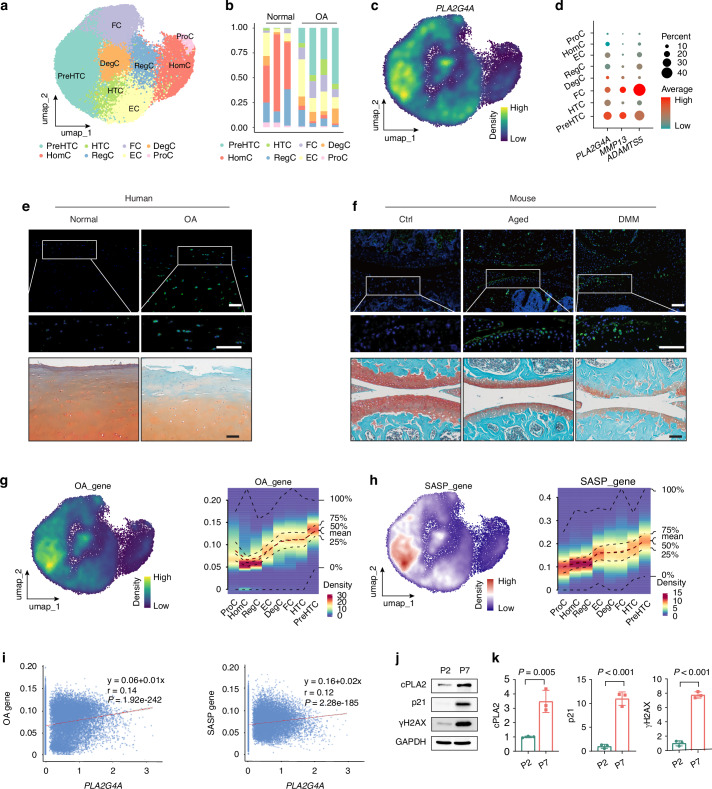
cPLA2 is predominatly expressed in preHTCs and up-regulated in OA cartilage. **a** Unbiased clustering of scRNA-seq data from human nonarthritic healthy (*n* = 3) and OA (*n* = 4) cartilage revealed 8 distinct cell clusters. **b** Stacked bar plot showing the proportion of chondrocyte subpopulations in human nonarthritic (*n* = 3) and OA (*n* = 4). **c** Density scatter plot of *PLA2G4A* expression visualized on the UMAP of chondrocyte. **d** Dot plot illustrating the expression patterns of *PLA2G4A*, *MMP13* and *ADAMTS5* across diverse chondrocyte subpopulations. **e** Representative immunofluorescence staining images of cPLA2 and Safranin O/Fast Green staining of human nonarthritic and OA cartilage. Scale bar = 100 μm. **f** Representative immunofluorescence staining images of cPLA2 and Safranin O/Fast Green staining of knee joints from ctrl, aged (18-month-old), and DMM mice (*n* = 6). Scale bar = 100 μm. **g**, **h** Density Scatter plot and density heatmap showing the distribution of OA-related (**g**) and SASP-related (**h**) gene sets across single-cell transcriptomic data. **i** Scatter plots depicting the relationships between *PLA2G4A* expression (x-axis) and the enrichment of OA-related genes (left) or SASP-related genes (right) across single cells. **j**, **k** Western blot analysis and quantification of cPLA2, p21, and γH2AX expression in primary human OA chondrocytes at passages 2 (P2) and 7 (P7) (*n* = 3). k, Data are mean±s.d., *P*-values by two-tailed unpaired Student’s *t*-test

To map the relative abundance and distribution of cPLA2 mRNA transcripts across distinct chondrocyte subpopulations, we analyzed our scRNA-seq dataset containing four human OA and three human normal cartilages (GSE169454).^37^ Unbiased clustering, guided by known cell-specific markers,^38^ identified eight distinct chondrocyte populations: homeostatic chondrocytes (HomCs), prehypertrophic chondrocytes (preHTCs), hypertrophic chondrocytes (HTCs), fibrochondrocytes (FCs), proliferative chondrocytes (ProCs), effector chondrocytes (ECs), regulatory chondrocytes (RegCs), and degradative chondrocytes (DegCs) (Fig. 1a and Fig. S1a). Notably, degenerative chondrocytes including preHTCs, HTCs, FCs and DegCs were significantly enriched in OA cartilage (Fig. 1b). The expression of *PLA2G4A* (encoding cPLA2) was predominantly localized to these degenerative chondrocytes, particularly preHTCs and FCs, the chondrocyte subtypes characterized by the expression of cartilage degradative genes such as *MMP13* and *ADAMTS5* (Fig. 1c, d). And compared with normal cartilage, the expression of *PLA2G4A* was increased in the course of OA cartilage (Fig. S1b, c). Consistent with mRNA expression data, immunofluorescence staining showed elevated cPLA2 protein levels in human OA cartilage, as well as in cartilage from mouse models of surgically induced OA and age-related degeneration (Fig. 1e, f and Fig. S1d).

Gene set enrichment analysis (Fig. 1g, h) demonstrated variability in cPLA2 expression across OA-associated and senescence-associated secretory phenotype (SASP)-associated gene sets, with peaks specifically highlighting preHTCs as key contributors to cartilage degenerative processes. From a correlation perspective, significant positive associations were observed between *PLA2G4A* expression and both OA-associated genes (*P* = 1.92 × 10^−^^242^, regression equation: y = 0.08 + 0.01x) and SASP-associated genes (*P* = 2.28 × 10^−185^, regression equation: y = 0.18 + 0.02x) (Fig. 1i), underscoring its regulatory role in cartilage degeneration and senescence processes. These findings position cPLA2 as a key regulator in OA pathogenesis, with its dual role in regulating key pathways contributing to chondrocyte senescence and cartilage degeneration. Chronic inflammatory is known to play important role in cartilage degeneration.^39^ Consistently, the inflammatory stimuli TNFα significantly enhanced cPLA2 expression at both mRNA and protein level in primary human OA chondrocytes (Fig. S1e–g). Additionally, treatment with etoposide (VP-16) or H_2_O_2_ to induce cellular senescence significantly elevated the senescence marker p21^40^ and increased cPLA2 mRNA and protein level (Fig. S1h–k). Furthermore, we induced primary human OA chondrocyte senescence by increasing passage number to passage 7, and found the senescent chondrocyte at passage 7 expressed higher level of cPLA2 and the senescence markers like p21 and γH2AX^40^ (Fig. 1j, k), suggesting cPLA2 as a senescence associated gene. Taken together, these results highlight cPLA2 as a previously unrecognized OA-associated gene, and critical mediator of senescence and cartilage degradation, making it a promising molecular target for further exploration in OA pathogenesis.

### Ablation of cPLA2 alters the expression of genes involved in chondrocyte metabolism and senescence

**Fig. 2:**
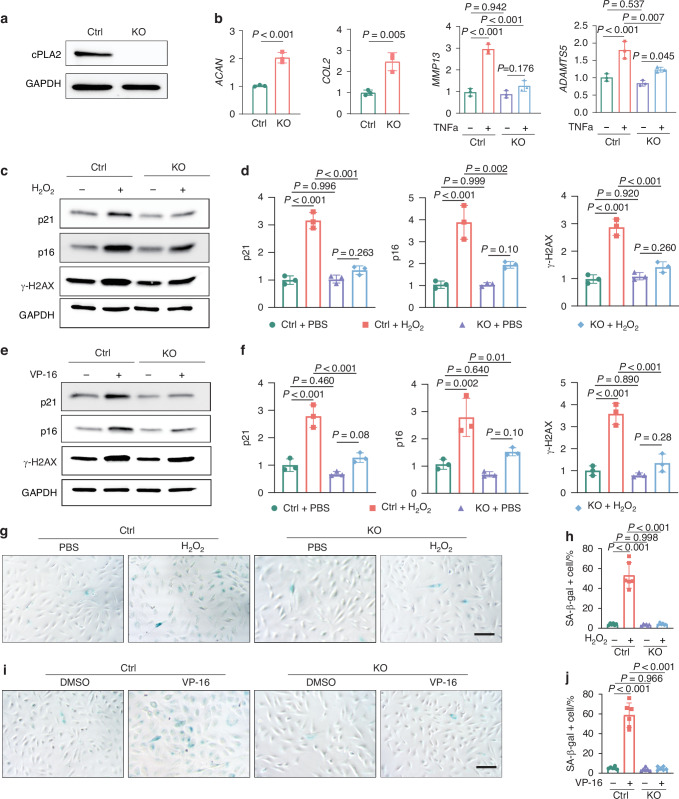
Knockout of cPLA2 modulates the expression of genes related to chondrocyte metabolism and senescence. **a** Western blotting to confirm the depletion of cPLA2 in cPLA2 knockout (KO) C28/I2 cells. **b** RT-qPCR analysis of *COL2*, *ACAN*, *MMP13* and *ADAMTS5* mRNA levels in WT and cPLA2 KO C28/I2 cells treated with or without 10 ng/mL TNFα (*n* = 3). **c**, **d** Western blot and quantification of p21, γH2AX, and p16 expression in WT and cPLA2 KO C28/I2 cells treated with 10 µmol/L H_2_O_2_ for 24 h (*n* = 3). **e**, **f** Western blot and quantification of p21, γH2AX, and p16 expression in WT and cPLA2 KO C28/I2 cells treated with 1 ng/mL VP-16 for 24 h (*n* = 3). **g**–**j** SA-β-galactosidase (SA-β-gal) staining and quantification of SA-β-gal positive cells of WT and cPLA2 KO C28/I2 cells treated with 10 µmol/L H_2_O_2_ (**g**, **h**) or 1 ng/mL VP-16 (**i**, **j**) for 48 h (*n* = 6). Scale bar = 100 μm. **b** Data are mean±s.d., *P*-values by two-tailed unpaired Student’s *t*-test (For ACAN and COL2); *P*-values by one way ANOVA with Bonferroni post hoc test (For ADAMTS5 and MMP13). **d**, **f**, **h**, **j** Data are mean±s.d., *P*-values by one way ANOVA with Bonferroni post hoc test

To directly evaluate the role of cPLA2 in chondrocyte regulation, we generated a cPLA2 knockout C28/I2 human chondrocyte cell line using the CRISPR/Cas9 technique (Fig. 2a). Knockout of cPLA2 significantly enhanced the expression of genes encoding anabolic molecules including *COL2* and *ACAN*, while inhibiting the expression of TNFα-induced catabolic markers such as *MMP13* and *ADAMTS5* (Fig. 2b). Moreover, cPLA2 knockout significantly decreased chondrocyte senescence phenotype induced by VP-16 or H_2_O_2_, evidenced by the inhibition of senescence markers p21, p16,^40^ and γ-H2AX (Fig. 2c–f). Senescence-associated β-galactosidase (SA-β-gal) staining further confirmed that cPLA2 knockout suppressed chondrocyte senescence (Fig. 2g–j). These findings highlight cPLA2 as a key regulator of chondrocyte anabolism, catabolism and senescence, suggesting its pivotal role in maintaining cartilage homeostasis and its potential as a therapeutic target in degenerative cartilage diseases.

### Pharmacological inhibition of cPLA2 modulates genes involved in chondrocyte metabolism and senescence

**Fig. 3:**
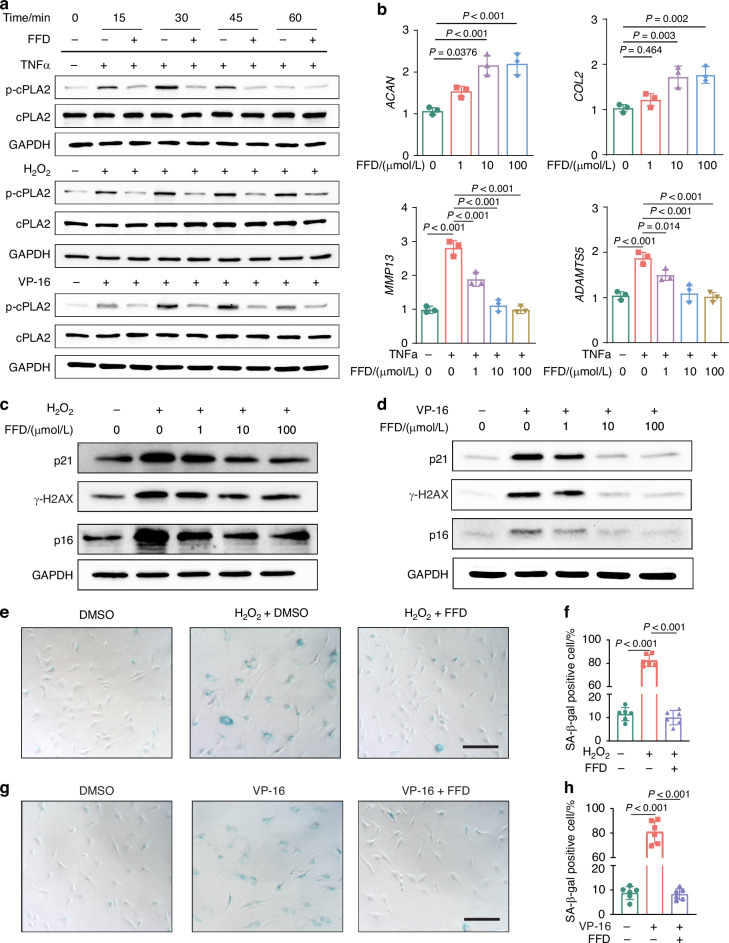
Pharmacologically inhibition of cPLA2 regulates the expression of genes related to chondrocyte metabolism and senescence. **a** Western blot analysis of p-cPLA2 and cPLA2 expression in primary human OA chondrocytes at various time points (15 min, 30 min, 45 min and 60 min) after stimulation with 10 ng/mL TNF-α, 10 µmol/L H_2_O_2_, 1 ng/mL VP-16, with or without 10 μmol/L FFD (*n* = 3). **b** mRNA levels of *COL2*, *ACAN* in human OA chondrocyte treated with varying concentrations of FFD for 24 h, assayed by qRT-PCR analysis. Additionally, mRNA levels of *MMP13*, *ADAMTS5* in chondrocyte treated with 10 ng/mL TNFα and varying concentrations of FFD for 24 h, assayed by qRT-PCR analysis (*n* = 3). **c** Western blot analysis of p21, γH2AX, and p16 expression in primary human OA chondrocytes treated with 10 µmol/L H_2_O_2_ and different concentrations of FFD (*n* = 3). **d** Western blot analysis of p21, γH2AX, and p16 expression in primary human OA chondrocytes treated with 1 ng/mL VP-16 and carying concentrations of FFD (*n* = 3). **e**–**h** SA-β-gal staining and quantification of SA-β-gal positive cells of primary human OA chondrocytes treated with 10 µmol/L H_2_O_2_ (**e**, **f**), or 1 ng/mL VP-16 (**g**, **h**) with/without 10 μmol/L FFD for 48 h (*n* = 6). Scale bar = 100 μm. **b**, **f**, **h** Data are mean±s.d., *P*-values by one way ANOVA with Bonferroni post hoc test

Our previous study identified FFD as an inhibitor of TNFα/cPLA2 signaling in macrophages, offering a novel strategy for targeting this critical inflammatory pathway. Specifically, FFD binds to the catalytic domain 2 of cPLA2 and inhibits its phosphorylation at Ser-505, which is required for its enzymatic activity.^35^ Building on this finding, we set to investigate whether FFD could be used a pharmacological inhibitor of cPLA2 through similar mechanism in chondrocytes. Both the inflammatory stimuli TNFα and senescence inducers VP-16 or H_2_O_2_ phosphorylated cPLA2 at Ser-505, and FFD significantly reduced the phosphorylation in human primary OA chondrocytes (Fig. 3a). Furthermore, FFD enhanced the expression of anabolic genes in a dose-dependent manner and suppressed TNFα-induced expression of catabolic genes (Fig. 3b). Similar to the effects observed with cPLA2 deletion, FFD effectively inhibited the expression of senescence markers such as p21, p16 and γ-H2AX induced by VP-16 or H_2_O_2_ in a dose-dependent manner (Fig. 3c, d, Fig. S2). SA-β-gal staining demonstrated that FFD significantly reduced number of SA-β-gal positive chondrocyte induced by H_2_O_2_ or VP-16 (Fig. 3e–h). More importantly, FFD-mediated protective effects in term of enhanced anabolism, inhibited inflammatory cytokine-induced catabolism, and suppressed cellular senescence, were largely abolished in cPLA2 knockout chondrocytes (Fig. S3). Collectively, these findings provide strong evidence that FFD directly targets cPLA2 and inhibits its phosphorylation, thereby modulating the expression of genes related to chondrocyte anabolism, catabolism, and senescence, highlighting its potential as a therapeutic agent in OA, particularly by targeting cPLA2 to mitigate inflammation- and senescence-driven cartilage degeneration.

### Deletion of cPLA2 protects against both surgically-induced and age-associated OA

**Fig. 4:**
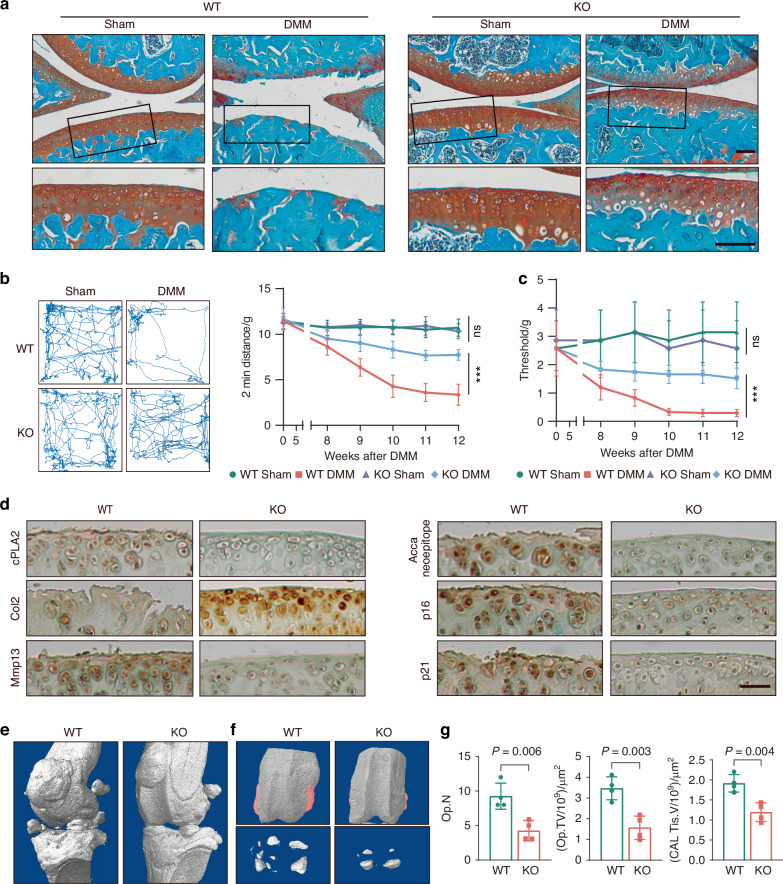
Deletion of cPLA2 protects against surgically-induced OA. **a** Representative Safranin O/Fast Green stained images of knee joints from WT and cPLA2 KO male mice (*n* = 7) at 12 weeks after DMM or sham surgery. Scale bar = 100 μm. **b**, **c** 2 min travel distance (**b**) and von Frey pain assay (**c**) in WT and cPLA2 KO mice at the indicated time after DMM surgery (*n* = 7). **d** Representative immunohistochemical staining images of cPLA2, Col2, Mmp13, aggrecan neoepitope, p16, and p21 in knee joint sections from each group (*n* = 7). Scale bar = 50 μm. **e** 3D micro-CT images of pathological changes in mouse knee joints 12 weeks post-surgery (*n* = 4). **f** 3D micro-CT images of osteophyte formation, calcified meniscus and synovial tissue, with red-marked regions indicating osteophytes (*n* = 4). **g** Quantification of osteophyte number (Op.N, I), size (Op.TV, J), and the volume of calcified meniscus and synovial tissue (Cal Tis.V, K). **g** Data are mean±s.d., *P*-values by two-tailed unpaired Student’s *t*-test

Given that cPLA2 is identified as an OA-associated gene that regulates chondrocyte homeostasis, we therefore explore its role in OA development using both surgically-induced destabilization of the medial meniscus (DMM) and naturally occurring age-associated OA models. Histological analysis revealed that deletion of cPLA2 significantly attenuated cartilage loss and reduced the Osteoarthritis Research Society International (OARSI) score in DMM OA model (Fig. 4a). Deletion of cPLA2 markedly reduced subchondral bone plate thickening, synovitis and osteophyte formation compared to WT control mice (Fig. S4a–c). To assess whether deletion of cPLA2 also alleviates OA-associated pain, we performed locomotor activity using an open field test and mechanical allodynia with von Frey testing. cPLA2 knockout (KO) mice demonstrated markedly increased movement distance and reduced mechanical allodynia throughout the three-month post-surgery period (Fig. 4b, c). Immunohistochemical staining further revealed that cPLA2 knockout largely prevented the loss of Col2 in cartilage 12 weeks after DMM surgery, suggesting that the absence of cPLA2 plays a protective role in maintaining cartilage extracellular matrix composition (Fig. 4d and Fig. S4d). Additionally, markers of chondrocyte catabolism such as MMP13 and aggrecan neoepitope, along with senescence marker including p16 and p21 were significantly reduced by cPLA2 deletion in cartilage of OA mice model (Fig. 4d and Fig. S4d). Consistent with histological analysis, micro-CT analysis of undecalcified joint samples revealed substantial osteophyte formation in WT mice 12 weeks post-DMM surgery. This was significantly inhibited by cPLA2 depletion (Fig. 4e, f). Additionally, changes in calcified meniscus and synovial tissue volume mirrored the trends observed in osteophyte formation (Fig. 4g).

**Fig. 5:**
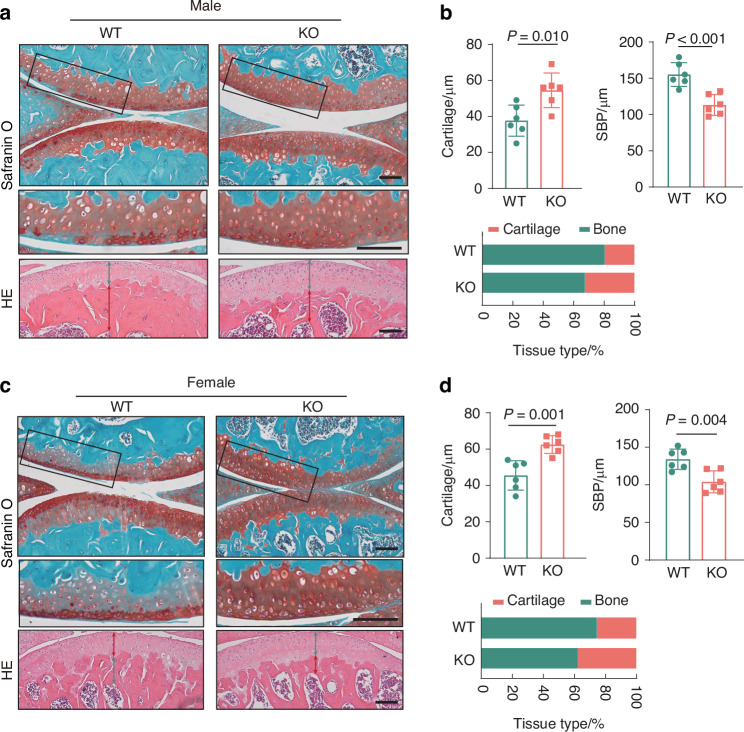
Deletion of cPLA2 protects against age-associated OA. **a** Safranin O/Fast Green, and H&E staining in knee joint section collected from WT and cPLA2 KO male mice at age 18 months. Scale bar = 100 µm. **b** Quantitation of the cartilage thickness, SBP thickness and the composition of the articular cartilage of WT and cPLA2 KO male mice based on HE staining (green: cartilage; red: bone). (*n* = 6). **c** Safranin O/Fast Green, and H&E staining in knee joint section collected from WT and cPLA2 KO female mice at age 18 months. Scale bar = 100 µm. **d** Quantitation of the cartilage thickness, SBP thickness and the composition of the articular cartilage of WT and cPLA2 KO female mice based on HE staining (green: cartilage; red: bone). (*n* = 6). **b**, **d** Data are mean±s.d., *P*-values by two-tailed unpaired Student’s *t*-test

In young adult mice (3 months old), Safranin O and H&E staining revealed no significant differences between cPLA2 knockout and WT littermates in proteoglycan content, cartilage thickness and subchondral bone plate thickness (Fig. S5a, b). However, in aged mice (18 months old), cPLA2 deletion significantly reduced OA like phenotype including proteoglycan loss, articular cartilage thinning and subchondral bone plate thickening, which were observed in age matched WT mice (Fig. 5a, b). Immunohistochemical staining further revealed that cPLA2 depletion could largely prevented reduction in Col2 levels in cartilage of 18 months old mice (Fig. S5c, d). Additionally, markers of chondrocyte catabolism such Mmp13 and aggrecan neoepitope, along with senescence markers including p16 and p21 were significantly inhibited by cPLA2 deletion (Fig. S5c,d). We also found that the knockout of cPLA2 exhibited a similarly significant cartilage-protective effect in female mice (Fig. 5c, d). These findings highlight the critical role of cPLA2 in OA pathogenesis, implicating it as a key player of cartilage degeneration, subchondral bone remodeling, and OA-associated pain. Targeting cPLA2 holds potential as a therapeutic strategy for managing both surgically-induced and age related OA.

### cPLA2 inhibition protects against OA in a surgically induced model

**Fig. 6:**
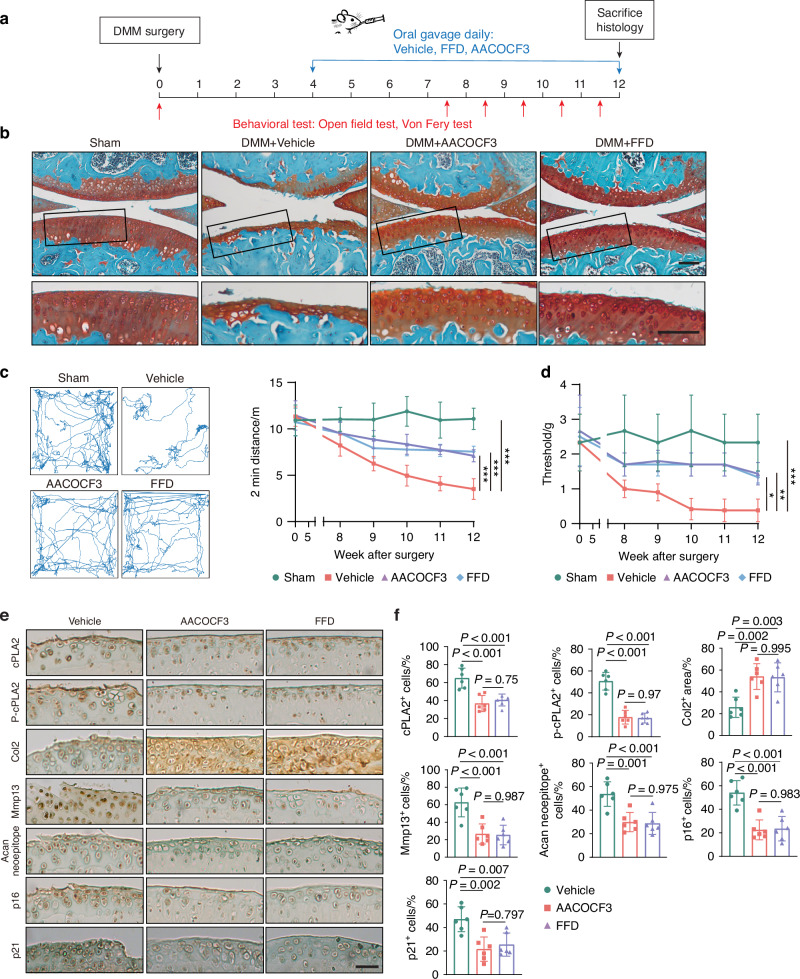
cPLA2 inhibition protects against OA in a surgically induced model. **a** Schematic of the experimental outline. 12-week-old male WT mice underwent DMM or sham surgery. Starting at 4 weeks post-surgery, the mice were treated daily with either 10 mg/kg body weight FFD or 10 mg/kg body weight AACOCF3 for a duration of eight weeks (*n* = 6). **b** Representative Safranin O/Fast Green stained images of knee joints treated with/without FFD or AACOCF3 at 12 weeks post-surgery (*n* = 6). Scale bar = 100 μm. **c**, **d** 2 min travel distance (**c**) and von Frey pain assay (**d**) in mice with varying treatment at the indicated time points after surgery (*n* = 6). **e** Representative immunohistochemical staining images of *p*-cPLA2, cPLA2, Col2, Mmp13, aggrecan neoepitope, p16, and p21 in knee joint sections from each group (*n* = 6). Scale bar = 50 μm. **f** Quantification of positive staining based on IHC images in (**e**) (*n* = 6). **c**, **d**, **f** Data are mean±s.d., *P*-values by one way ANOVA with Bonferroni post hoc test. *, *P* < 0.05, **, *P* < 0.001, ***P < 0.001

We next explored the potential of pharmacological inhibition of cPLA2 using the FDA-approved FFD for treating OA. WT mice subjected to DMM surgery were treated orally with or without FFD or AACOCF3^41^ (a known cPLA2-specific inhibitor used as a positive control) starting 4 weeks post-operatively for 8 weeks (Fig. 6a). Similar to genetic cPLA2 deletion, pharmacological inhibition of cPLA2 with either FFD or AACOCF3 significantly reduced cartilage loss, synovitis, subchondral bone thickness and osteophyte formation relative to vehicle-treated controls (Fig. 6b and Fig. S6a–c). Additionally, both FFD and AACOCF3 significantly alleviated OA-associated pain with increased movement distance and higher paw withdrawal threshold (Fig. 6c, d).

As expected for functional inhibitors, both compounds directly decreased p-cPLA2 protein levels. Interestingly, total cPLA2 expression was also reduced. This finding is consistent with our earlier observations: OA progression drives up cPLA2 levels, whereas inhibition of cPLA2 both prevents OA development and leads to an overall decrease in cPLA2 expression (Fig. 6e, f). Immunohistochemical staining showed that FFD or AACOCF3 treatment significantly increased Col2 levels, while decreased Mmp13 and aggrecan neoepitope levels (Fig. 6e, f). Additionally, both treatments markedly decreased the expression of senescence markers p16 and p21 in chondrocytes (Fig. 6e, f). Collectively, these results suggest that pharmacological cPLA2 inhibition effectively protect against OA progression in the surgery-induced OA mouse model, and that the FDA-approved drug FFD could potentially be repurposed for OA treatment.

To further confirm that the therapeutic effects of FFD on OA through targeting cPLA2, we performed DMM surgery in cPLA2 KO mice followed by treatment with or without FFD. Histological analysis revealed that FFD treatment in cPLA2 KO mice did not provide additional protective effects in mitigating cartilage loss, reducing OARSI scores, suppressing osteophyte formation, or preventing subchondral bone plate thickening (Fig. S6d, e). These findings align with structural changes observed, as FFD treatment in cPLA2 KO mice did not result in increased overall movement distance or reduced mechanical allodynia throughout the 3-month period following DMM surgery (Fig. S6f–h). Furthermore, immunohistochemical staining confirmed that anabolic markers, catabolic markers, and chondrocyte senescence markers remained largely unchanged with FFD treatment in cPLA2 KO mice (Fig. S6i, j). To be noted, FFD treatment provided no additional protective effects against OA progression or OA-associated behavioral changes in cPLA2 KO mice compared to vehicle treated controls, indicating that FFD’s therapeutic effects in OA primarily relies on its ability to target cPLA2.

### Genetic deletion of cPLA2 alleviates both surgically-induced and age-associated IVDD

The vertebral endplate is a biolayer structure consisting of a cartilage layer as a bony layer, playing a critical role in the pathogenesis of IVDD and the onset of low back pain.^42,43^ Although it was reported that FFD protects against disc degeneration through TNFα signaling in a rat needle puncture IVDD model,^44^ the specific target underlying its therapeutic effects remains unknown. To map the relative abundance and distribution of cPLA2 mRNA transcripts across distinct chondrocyte subpopulations of vertebral endplate, we analyzed the scRNA-seq dataset containing two normal human vertebral endplate samples^45^ (GSE160756). Similar to articular cartilage from human knee joints, unbiased clustering, guided by known cell-specific markers, identified eight distinct populations: HomCs, preHTCs, HTCs, FCs, ProCs, ECs, RegCs, and DegCs (Fig. S7a). The expression of *PLA2G4A* was also predominantly localized to degenerative chondrocytes, particularly preHTCs and FCs of end plate, characterized by the expression of cartilage degradative genes such as *MMP13* (Fig. S7b–d). To further investigate whether FFD exerts its protective effects through targeting cPLA2 and evaluate the potential of cPLA2 as a therapeutic target for IVDD treatment, we utilized a lumbar spine instability (LSI) model in mice^46^ (Fig. S8a). Safranin O staining revealed a substantial loss of cartilage portion of the endplate following the induction of the LSI model, which was effectively attenuated in cPLA2 KO mice (Fig. S8b, c). Similarly, H&E staining indicated significant degeneration in WT mice, as evidenced by increased histological scores,^47^ while cPLA2 KO mice exhibited notable preservation of disc integrity. Immunochemistry staining supported these findings, showing a significant reduction in Col2 level in endplate 12 weeks after LSI surgery in WT mice compared to cPLA2 KO mice. Additionally, markers of chondrocyte catabolism, such as Mmp13, along with senescence markers p16 and p21, were significantly downregulated in the endplates of cPLA2 KO mice compared to WT mice (Fig. S8d, e). Notably, FFD treatment did not further enhance these effects in cPLA2 KO mice (Fig. S9). Collectively, these findings suggest cPLA2 could be a potential target for IVDD treatment and indicate that FFD protects against IVDD also through the inhibition of cPLA2.

Intervertebral discs undergo age-related changes that lead to degeneration and pain, with cellular senescence playing a critical role in the progression of IVDD.^48^ To evaluate the potential of cPLA2 as a therapeutic target for age-associated IVDD, we examined 18-month-old aged cPLA2 knockout and WT mice, comparing their cartilage proportion of end plate and intervertebral disc structure to those of 3-month-old young adult mice. At 3 months of age, no significant differences in the intervertebral discs were observed between cPLA2 KO and WT mice (Fig. S10a, b). However, by 18 months, histological analysis using Safranin O and H&E staining revealed significantly less severe disc degeneration in cPLA2 KO mice compared to their WT counterparts. WT mice exhibited hallmarks of IVDD, including higher end-plate scores and instances of nucleus pulposus breakthrough into the annulus fibrosus. In contrast, cPLA2 KO mice exhibited reduced cartilage loss in the endplate, lower disc degeneration and preservation of disc integrity (Fig. S10c, d). Molecular marker analysis further support these observation by demonstrating that cPLA2 knockout mice has higher levels of Col2, and lower levels of Mmp13 as well as p16 and p21 in endplate cartilage (Fig. S10e, f). In our analysis of tissues collected from aged female subjects, we observed that cPLA2 knockout conferred significant protective effects, similar to those observed in aged male mice (Fig. S10g, h). These findings suggest that cPLA2 contributes to age-related degenerative changes in endplate cartilage and intervertebral discs, and its genetic deletion provides protection against IVDD in aging mice. This study highlights cPLA2 as promising therapeutic target for addressing age-associated IVDD and its related complications.

## Discussion

The study reveals cPLA2 as a critical regulator of cartilage degeneration and cellular senescence implicated in the pathogenesis of cartilage degenerative diseases including OA and IVDD, and highlights the therapeutic potential of FFD for treating these degenerative conditions through targeting cPLA2. Single-cell RNA sequencing showed that cPLA2 is enriched in degenerative chondrocytes, particularly preHTCs and FCs, characterized by elevated cartilage degeneration and SASP-related genes,^49^ underscoring its critical role in amplifying inflammatory signals, promoting matrix degradation and cellular senescence. Consistently, scRNA-seq with human normal endplate cartilage also revealed the presence of similar chondrocyte landscape in endplate cartilage of intervertebral disc, and cPLA2 is predominantly expressed in preHTCs and FCs. Furthermore, cPLA2 expression was upregulated in response to inflammatory and senescence inducing stimuli in primary human OA chondrocytes, linking it to chronic inflammation and senescence, two critical drivers of OA progression. Comprehensive in vitro and in vivo studies provided compelling evidence that cPLA2 play important regulatory role in anabolic and catabolic pathways, cPLA2 inhibition reduced the expression of inflammatory and degradative markers while enhancing anabolic activities.^44,50,51^

Similar to inhibiting TNFα-induced inflammation, cPLA2 plays a pivotal role in regulating chondrocyte senescence, a key hallmark of age-related OA and IVDD progression. Chondrocyte senescence is characterized by irreversible cell cycle arrest, metabolic dysfunction, and the secretion of pro-inflammatory and catabolic factors collectively referred to as the SASP.^52,53^ cPLA2 contributes to this process by catalyzing the release of arachidonic acid, the precursor of pro-inflammatory lipid mediators and its downstream eicosanoids such as prostaglandins and leukotrienes,^54–56^ activate signaling pathways (such as NF-κB and MAPK) that enhance the expression of catabolic genes and senescence-associated genes while simultaneously suppressing anabolic processes crucial for cartilage maintenance.^57,58^ Although the precise molecular connections remain to be fully elucidated - an important area for future research - this dual role accelerates the degradation of key extracellular matrix components, such as Col2 and aggrecan, while simultaneously promoting the accumulation of senescent cells within cartilage and intervertebral discs. Together, these processes further drive the degenerative cycle characteristic of age-related OA and IVDD. Targeting cPLA2 offers a promising therapeutic approach to mitigate chondrocyte senescence, reduce SASP-associated inflammation, and preserve tissue integrity in both age-related OA and IVDD.

Building on the identification of cPLA2 as a therapeutic target, we tested FFD, an FDA-approved drug known to inhibit cPLA2 activity.^35^ FFD effectively attenuated TNFα-induced catabolic responses and senescence in chondrocytes, mirroring the effects of cPLA2 deletion. Importantly, the protective effects of FFD were abolished in cPLA2 knockout chondrocytes, confirming its mechanism of action through cPLA2 inhibition. In a surgically induced OA model, pharmacological inhibition of cPLA2 with FFD significantly reduced cartilage degeneration, subchondral bone changes, osteophyte formation, and OA-associated pain, comparable to the effects observed in cPLA2 knockout mice. These results establish FFD as a potential repurposed therapeutic agent for OA, with its efficacy directly linked to targeting cPLA2.

Our findings extend the role of cPLA2 to IVDD, where its deletion protected against both surgically induced and age-associated disc degeneration. Histological and molecular analyses demonstrated that cPLA2 knockout preserved disc integrity, reduced cartilage loss in the endplate, and suppressed senescence and catabolic markers in both surgically induced and aged mouse models. FFD similarly conferred protection against disc degeneration through cPLA2 inhibition, but its effects were not additive in cPLA2-deficient mice, further supporting the specificity of cPLA2 as its primary target.

Significant efforts have been dedicated over the past few decades to developing potent cPLA2 inhibitors. However, the clinical application of these compounds has faced major challenges, including toxicity and poor intestinal absorption, despite numerous cPLA2 inhibitors undergoing clinical trials. Our discovery that the widely used over-the counter drug FFD^59–61^ effectively targets and inhibits cPLA2, coupled with its demonstrated efficacy in animal models of OA and IVDD, offers a promising avenue to overcome these barriers and advance the development of cPLA2-targeted therapies. The identification of FFD as a clinically available inhibitor of cPLA2 offers an immediate opportunity for translational research and potential repurposing for treating OA and IVDD.

It is also important to note that multiple pathways — including Wnt/β-catenin signaling, TGF-β signaling, and mechanical stress responses — have been implicated in the progression of osteoarthritis (OA). Although cPLA2 plays a significant role, it is unlikely to act as the sole driver of disease, given the presence of compensatory and redundant mechanisms. Instead, cPLA2 likely functions as a key amplifier of pathological signaling, and its inhibition can substantially shift the balance toward tissue protection, even though it does not fully negate the contributions of other disease-promoting pathways. While our data suggest a chondroprotective effect, we cannot exclude the potential involvement of other joint-resident cell types, such as synoviocytes and immune cells. Future studies using conditional knockout models or localized delivery approaches will be critical to precisely delineate the specific cell populations involved. Given the inherent complexity of in vivo conditions, further research is essential to clarify the interplay among these signaling pathways and ultimately develop more effective and efficient therapeutic strategies.

It is also important to note that multiple pathways - including Wnt/β-catenin signaling, TGF-β signaling, and mechanical stress responses - have been implicated in the progression of OA.^62–64^ Although cPLA2 plays a significant role, it is unlikely to act as the sole driver of disease, given the presence of compensatory and redundant mechanisms. Instead, cPLA2 likely functions as a key amplifier of pathological signaling, and its inhibition can substantially shift the balance toward tissue protection, even though it does not fully negate the contributions of other disease-promoting pathways. Additionally, while our data suggest a chondroprotective effect, we cannot exclude the potential involvement of other joint-resident cell types, such as synoviocytes and immune cells. Future studies using conditional knockout models or localized delivery approaches will be critical to precisely delineate the specific cell populations involved. Given the inherent complexity of in vivo conditions, further research is essential to clarify the interplay among these signaling pathways and ultimately develop more effective and efficient therapeutic strategies.

In conclusion, the identification of cPLA2 as a critical regulator in the pathogenesis of degenerative disease including OA and IVDD, opening exciting opportunities for targeted interventions. The dual action of FFD in alleviating inflammation and senescence while preventing cartilage degeneration highlights its potential as a chondroprotective agent. Future research should explore the broader applicability of FFD in other cPLA2-mediated degenerative diseases and investigate its long-term efficacy and safety in clinical settings. This study provides compelling evidence for the critical role of cPLA2 in degenerative disease including OA and IVDD and positions FFD as a promising therapeutic candidate. By targeting cPLA2, FFD not only improves structural and symptomatic outcomes associated with OA and IVDD but also offers insights into the molecular mechanisms underlying cartilage degeneration.

## Materials and Methods

### Mice

All animal experiments in this study were approved by the Institutional Animal Care and Use Committee (IACUC) of Yale University School of Medicine and conducted in compliance with the relevant ethical regulations for animal testing and research. Mice were housed in a specific pathogen-free environment under a 12 h light-dark cycle, with free access to food and water.

cPLA2^+/–^ heterozygous mice, initially generated in Dr. Joseph V. Bonventre’s lab at Brigham and Women’s Hospital, were generously provided by Dr. Naikui Liu’s lab at Indiana University School of Medicine. These mice are on a C57BL/6 background, and cPLA2 knockout (KO) mice were produced by crossing heterozygotes. Wild-type (WT) littermates served as controls. Genotyping was performed by PCR following previously published protocols. Unless otherwise stated, all mice used in experiments were on a C57BL/6 J background, matched by sex and age.

### Aging-associated and surgically-induced DMM mouse OA models

To study aging-associated osteoarthritis (OA), WT and cPLA2 KO mice were kept under normal conditions until 3 or 18 months of age, at which time knee joints were evaluated for spontaneous OA onset. For the surgically induced destabilization of the medial meniscus (DMM) model, 12-week-old cPLA2 KO and age-matched WT male C57BL/6 J mice underwent DMM surgery. To establish the surgically induced DMM model,^65^ after ketamine and xylazine anesthesia, the medial meniscotibial ligament in the right knee was sectioned with a blade to destabilize the medial meniscus. Sham DMM operations were performed by opening the joint capsule of the right knee of independent mice. To assess the therapeutic effects of fexofenadine (FFD) or AACOCF3 (a known cPLA2 inhibitor) across different genetic backgrounds, mice received oral gavage of either vehicle (5% DMSO + 10% ethanol), AACOCF3 (10 mg/kg, sc-201412A, Santa Cruz), or FFD (10 mg/kg, S3208, Selleckchem) for 8 weeks, starting 28 days post-DMM surgery. Knee joints were harvested 12 weeks later for histology.

### Aging-associated and surgically-induced LSI surgery

Similarly, for aging-associated intervertebral disc degeneration (IVDD) study, WT and cPLA2 KO mice were maintained under normal conditions to 3 or 18 months of age, after which disc tissues were examined for spontaneous IVDD degeneration. In addition, a lumbar spine instability (LSI) surgical procedure was performed to establish an IVDD model.^46^ Under anesthesia, a midline incision was made over the lumbar region, and the skin, supraspinous and interspinous ligaments, and spinous processes spanning L3 to L5 were removed. The wound was then closed, and topical antibiotics were applied to prevent postoperative infection. In sham-operated controls, only the posterior paravertebral muscles were detached from the L3–L5 vertebrae, without further disruption of ligaments or bone. Intervertebral discs at the L4–L5 level were selected for subsequent analysis. Beginning 28 days post-LSI surgery, mice in the “cPLA2 KO + FFD” group received oral gavage of FFD (10 mg/kg/d) for 8 weeks. “WT + Vehicle” and “cPLA2 KO + Vehicle” received vehicle at equivalent volumes. Mice were euthanized at 12 weeks post-surgery, with 6 animals in each group (WT + Vehicle, cPLA2 KO + Vehicle, cPLA2 KO + FFD).

### Human subjects research

All human experiments were conducted in accordance with protocols approved by Yale University Institutional Review Board (IRB Study Number 2000035962). Human cartilage samples were collected from patients undergoing total knee replacement at Bridgeport Hospital Milford Campus. Cartilage specimens were sectioned into 1 mm fragments, then digested overnight with 0.25% collagenase II. On the following day, chondrocytes were filtered from the residual tissue and cultured in DMEM supplemented with 10% fetal bovine serum, 50 U/mL penicillin, and 0.05 mg/mL streptomycin. Primary chondrocytes were treated with or without the indicated compounds.

### Single-cell Sequencing and data analysis

We performed down-sampling analysis across samples based on the mapped barcoded reads per cell for each sample, ultimately generating an aggregated matrix.^66^ Cell quality filtering criteria included a range of 200–6 000 expressed genes and a mitochondrial UMI rate below 20%. Data normalization and regression were conducted using the Seurat package (version 5.1.0, https://satijalab.org/seurat/), based on UMI counts per sample and mitochondrial percentage, to obtain scaled data. ScRNA-seq data from different samples were merged into a single Seurat object, normalized using the NormalizeData function, and scaled on the 2 000 most variable features using the FindVariableFeatures and ScaleData functions. Principal component analysis (PCA) was conducted with the RunPCA function, followed by batch effect correction using the RunHarmony function. Using a graph-based clustering approach, we obtained unsupervised cell cluster results. Marker genes were calculated with the FindAllMarkers function, employing the Wilcoxon rank-sum test under the following criteria: (1) log fold change (lnFC) > 0.25, (2) *P*-value < 0.05, and (3) minimum percentage (min.pct) > 0.1. To refine cell type identification, clusters representing the same cell type were selected for further UMAP analysis, graph-based clustering, and marker analysis.

### Gene set enrichment analysis

Gene set enrichment analysis was conducted using the irGSEA R package (version 3.3.0). Specifically, density scatterplots and density heatmaps were generated via the Ucell method to illustrate the expression of OA- and senescence-associated secretory phenotype (SASP)-related gene sets across all cell types (Supplementary Table 1). The Pearson correlation coefficient (r) was then used to evaluate the linear relationship between cPLA2 expression and the enrichment scores.

### Generation of cPLA2 KO chondrocytes by CRISPR-Cas9

cPLA2 KO chondrocytes were created following a previously reported CRISPR-Cas9 protocol.^67^ Briefly, cPLA2 sgRNA (F: CACCGACAGAGCCATACTAAATCGT, R: AAACACGATTTAGTATGGCTCTGTC) was cloned into the lentiCRISPR V2 vector (Addgene #52961). HEK293T cells were co-transfected with this plasmid, psPAX2 (Addgene #12260), and pMD2.G (Addgene #12259) to produce lentivirus. Cells co-transfected a non-targeting, random sgRNA sequence served as the control. C28/I2 human chondrocytes were infected with the resulting lentivirus for 18 hours, then selected with 2 µg/mL puromycin (Gibco, Cat. A1113803) for 2 days.

### Cell culture and treatment

C28/I2^68^ and primary human chondrocytes were cultured in Dulbecco’s Modified Eagle’s Medium (DMEM) supplemented with 10% fetal bovine serum, 50 U/mL penicillin, and 0.05 mg/mL streptomycin. C28/I2 Human Chondrocyte Cell Line was derived from juvenile costal cartilage and immortalized by transduction of vectors encoding simian virus 40 infection, widely used as a model cell line for studying normal and pathological cartilage repair and senescence mechanisms related to chondrocyte biology and physiology.^69–72^ Inflammation was induced in chondrocytes using TNFα, while senescence was promoted using VP-16 and H_2_O_2_. For inhibitor treatments, cells were pretreated with or without FFD (S3208, Selleckchem) at a concentration of 10 μmol/L prior to drug incubation for specified culture durations, followed by analysis of anabolic and senescence markers.

### Quantitative Real-time PCR (qRT-PCR)

For qRT-PCR, RNA was extracted using a RNeasy Mini Kit (Qiagen, Cat. 74104). cDNA preparation and qRT-PCR analysis were conducted as previously described. All primer sequences of target genes are listed in Supplementary Table 2. The gene expression levels were calculated using the 2^−ΔΔCT^ method. The data were reported as fold increases compared with endogenous controls.

### Western blot analysis

Western blotting was performed by separating protein samples on an SDS-PAGE gel, followed by transfer to a nitrocellulose (NC) membrane (Millipore, Cat. HATF00010). After blocking with 5% non-fat milk in TBST for 1 hour at room temperature, membranes were incubated overnight at 4 °C with the specified primary antibodies, then with the corresponding secondary antibodies for 1 h at room temperature. Signals were detected using an enhanced chemiluminescence (ECL) substrate and visualized on a GelDoc system. Primary antibodies included p-cPLA2 (CST, Cat. 2832), cPLA2 (CST, Cat. 2831), p16 (CST, Cat. 18769), p21 (CST, Cat. 2947), phospho-Histone H2A.X (CST, Cat. 9718), and GAPDH (ProteinTech, Cat. 60004-1-Ig).

#### SA-β-gal staining

SA-β-gal staining Kit (CST #9860) was used. Fixative solution was cultured after the intervention and left to fix at room temperature for 15 min. A suitable quantity of staining solution was obtained, cleaned with PBS, applied to the culture wells, and kept at 37 °C without the presence of CO_2_ overnight. The last step was to use a high-resolution microscope to capture pictures.

### Micro-CT

Knee joints from mice were collected 12 weeks post-surgery. Prior to histological processing, the paraformaldehyde-fixed knee joints of WT and cPLA2 KO mice were subjected to micro-CT imaging using a Scanco vivaCT40 cone-beam scanner (SCANCO Medical, Switzerland) at a resolution of 10.5 μm, with a 55 kVp source and 145 μAmp current. After image reconstruction and orientation, osteophyte development and subchondral bone plate thickness were quantified through 3D analysis using CT-Analyser (Bruker).

Total osteophyte volume was calculated by manual segmentation of a region of interest encompassing osteophytic tissue, identified by deviations from the cortical bone boundary, at intervals of at least every five slices with adaptive interpolation. Subchondral bone plate thickness in the medial tibia was measured using the largest-sphere fitting algorithm within a defined region of interest measuring 0.5 mm by 5 mm. Trabecular bone was excluded through manual segmentation performed every 10 slices. A Gaussian filter, despeckle function, and global thresholding were applied to all datasets prior to analysis.

### Histology and Immunohistochemistry (IHC)

Mice tissue samples were collected 12 weeks post-surgery. The tissues were decalcified in 10% w/v EDTA for 3 weeks, paraffin-embedded, and sectioned into serial 6 μm sagittal slices. Serial sections were stained with Safranin-O or H&E staining. Cartilage destruction was graded on Safranin-O stained sections by blinded observers using the OARSI histology scoring system (grade 0-6).^73^ Synovial inflammation was determined by H&E staining (grade 0-3)^74^ as previously described. Osteophyte development was evaluated and the thickness of the subchondral bone plate was measured^75^ on Safranin-O stained sections, and osteophyte maturity was scored (grade 0-3).^76^ The histological score of IVDD model was determined as previously described based on the H&E staining or Safranin O staining.^47^ Briefly, cellularity and morphology of the AF, NP, and the border between the AF and NP was determined in this grading system. Histological score analysis included five categories. Total grades ranged from 5 to 15, in which normal disc get one point in each category and most severe degeneration scored 15. Endplate score^77,78^ was determined based on assessments of cells, cartilage disorganization, cartilage cracks, microfractures, new bone formation, and bone sclerosis, with a total possible score ranging from 0 to 18. Tissue type % refers to the proportion of a specific tissue thickness (cartilage and subchondral bone) within the total analyzed tissue.

For IHC, deparaffinized and hydrated sections were incubated with 0.1% trypsin for 30 minutes at 37 °C, followed by treatment with 0.25 U/mL chondroitinase ABC (Sigma) and 1 U/mL hyaluronidase (Sigma) for 60 min at 37 °C. Sections were incubated overnight at 4 °C with primary antibodies against Col II (ProteinTech, Cat. 28459-1-AP), aggrecan neoepitope (Novus Biologicals, Cat. NB100-74350), MMP13 (ProteinTech, Cat. 18165-1-AP), cPLA2 (CST, Cat. 2831), p16 (CST, Cat. 18769), and p21 (Invitrogen, Cat. MA5-14949). Detection was performed with the Vectastain Elite ABC kit (Vector Laboratories, PK-6100), and positive signals were visualized using 0.5 mg/mL 3,3-diaminobenzidine in a 50 mmol/L Tris-HCl substrate (Sigma). Slides were counterstained with 1% methyl green, and images were acquired with a Zeiss Axioscope A1 microscope. Quantification of immunohistochemical staining was performed using ImageJ, applying global thresholding for comparable groups of IHC images.

### Safranin O staining

Paraffin-embedded knee and ankle sections were deparaffinized using a xylene and ethanol gradient. Sections were stained with 2% hematoxylin (23412, MilliporeSigma) for 5 minutes, followed by 1.0% Safranin O (S8884, Sigma-Aldrich) for 60 minutes, and counterstained with 0.02% Fast Green (F7258, Sigma-Aldrich) for 1 minute. The slides were then dehydrated, cover-slipped, and imaged using a light microscope (Axio Scope A.1, Carl Zeiss, LLC).

### Behavioral Assessment

The mechanical allodynia test was conducted using a calibrated set of von Frey filaments (North Coast Medical Inc., CA, USA).^79^ Before testing, mice were allowed to acclimate for 15 minutes on a wire mesh grid. Filaments with a force range typically used for mice (0.04–2.0 g, starting with 0.04 g) were applied to the plantar surface of the hind paw to determine the 50% force withdrawal threshold using the classical up-down iterative method, as previously described. A positive response was recorded if the mouse exhibited any nociceptive behavior, such as brisk paw withdrawal, licking, or shaking of the paw, either during filament application or immediately after its removal. All tests were conducted by an investigator blinded to the experimental groups.

Spontaneous behavior was assessed using the open field test. Briefly, mice were placed in a 40 × 40 × 40 cm polyvinyl chloride (PVC) box, which was cleaned before each test with 70% ethanol and wiped dry with a paper towel. Mice were placed in the center of the box and allowed to move freely for 3 minutes, while their movements were recorded by a camera. Behavioral tracking software was used to analyze travel distance and other movement parameters.

### Statistical analysis

All data are expressed as means ± s.d.; The number of mice per genotype is specified in the figure legends. Comparisons between two groups were performed using two-tailed unpaired Student’s *t-*tests. For comparisons among multiple groups, one-way ANOVA followed by a post hoc Bonferroni test was applied. A *P*-value of < 0.05 was considered statistically significant.

## Supplementary information

online supplemental file

## Data Availability

Single-cell RNA-seq data that support the findings of this study obtained from Gene Expression Omnibus (GEO) with the accession codes GSE169454 (https://www.ncbi.nlm.nih.gov/geo/query/acc.cgi?acc=GSE169454) and GSE160756 (https://www.ncbi.nlm.nih.gov/geo/query/acc.cgi?acc=GSE160756). All other relevant data from this study are available from the corresponding authors upon reasonable request.
