# Supplementary material for: Cytosolic phospholipase A2 as a therapeutic target for degenerative joint diseases
Source: Bone Res. 2025 Oct 15;13:86. doi: 10.1038/s41413-025-00470-9 (PMC12528370; doi:10.1038/s41413-025-00470-9)
Supplement: Supplementary file 1 — online supplemental file [file 41413_2025_470_MOESM1_ESM.docx]

**Cytosolic phospholipase A2 as a therapeutic target for degenerative joint diseases**

Guiwu Huang^1,2#^, Chaopeng He^1,3#^, Wenyu Fu^1^, Jingwei Bi^1^, Jianji Wang^1^, Daniel H Wiznia^1^, Chuanju Liu^1*^

1. Department of Orthopaedics and Rehabilitation, Yale University School of Medicine, New Haven, CT, USA
2. Department of Joint Surgery, The First Affiliated Hospital of Sun Yat-sen University, Sun Yat-sen University, Guangzhou, China.
3. Department of Orthopedics, The Second Xiangya Hospital of Central South University, Changsha, China.

*To whom correspondence should be addressed: Dr. Chuan-ju Liu, Charles W. Ohse Professor of Orthopaedics, Yale School of Medicine. 789 Howard Avenue, New Haven, CT 06519. Tel: 203-7855968; Email: chuan-ju.liu@yale.edu

#Contributed equally.

This Supplementary Materials file includes:

Figs. S1 to S10 (Pages 2-17)

References (Pages 18-19)

**
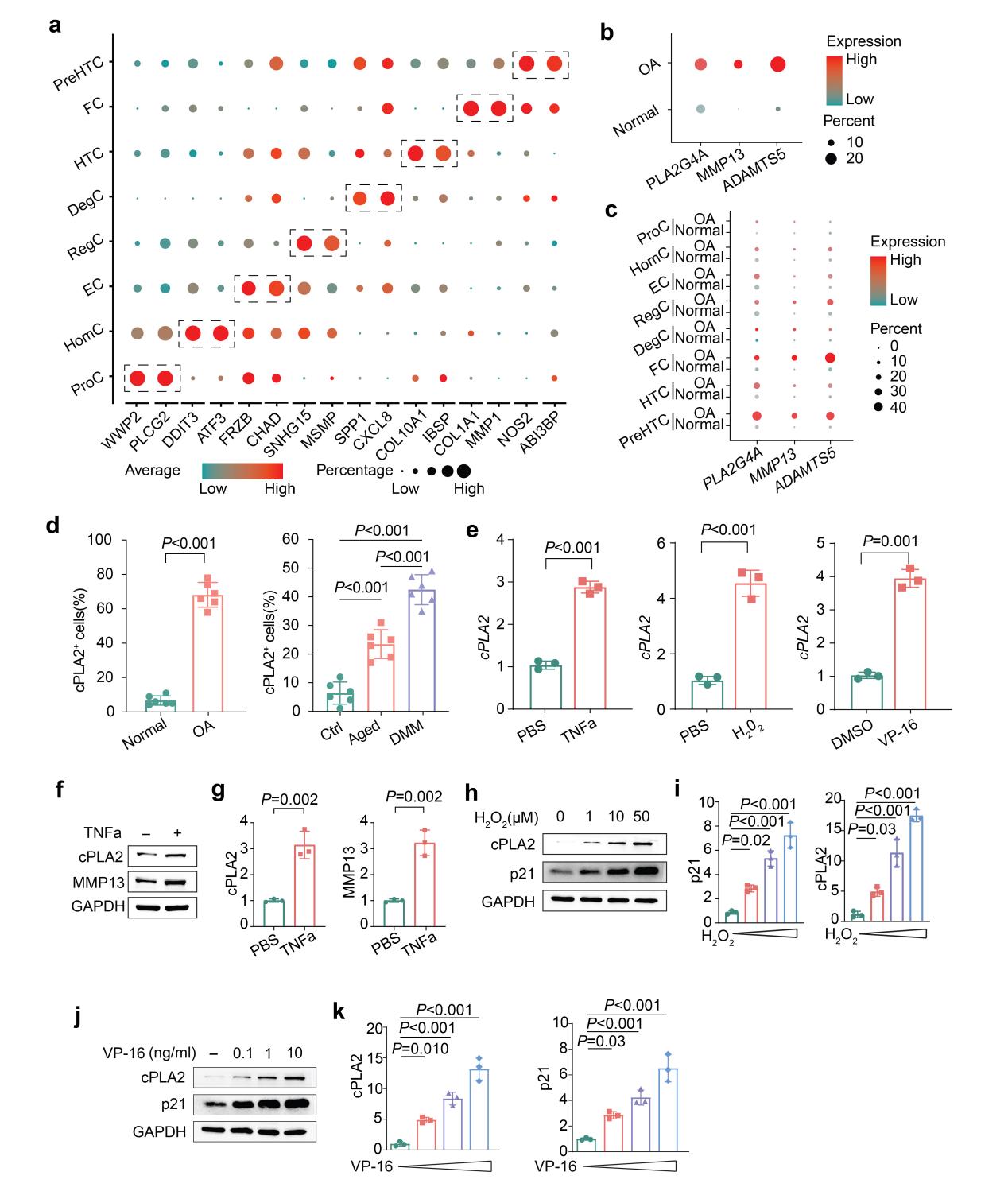
**

**Fig. S1. cPLA2 is upregulated in OA and senescent chondrocytes.** (a) Dot plot depicts the expression of feature genes across each chondrocyte cluster. (b) Dot plot illustrates the expression patterns of *PLA2G4A* across human normal and OA cartilage. The size of dots represents the percentage of cell expressions of the gene; color scale indicates the mean expression level. (c) Single-cell characterization of *PLA2G4A*, *MMP13* and *ADAMTS5* expression across varying chondrocyte populations in human normal and OA chondrocytes. Bubble plot visualization of *PLA2G4A*, *MMP13* and *ADAMTS5* expression across 8 different clusters cells identified in human normal and OA cartilage using scRNA-seq. (d) Quantification of positive staining based on representative immunofluorescence images of cPLA2 of cartilage from human or mice as shown in Fig. 1e, f) (n=6). (e) RT-qPCR analysis of *cPLA2* mRNA levels in primary human OA chondrocytes treated with 10 ng/mL TNF-α, 1 ng/mL VP-16 or 10µM H_2_O_2_ for 24 hours (n = 3).

(f,g) Western blot analysis (f) and quantification (g) of cPLA2 and MMP13 expression in primary human OA chondrocytes treated with 10 ng/mL TNF-α for 48 hours (n = 3). (h,i)Western blot analysis (h) and quantification (i) of cPLA2 and p21 expression in primary human OA chondrocytes treated with various concentrations of H_2_O_2_ for 24 hours (n = 3). (j,k) Western blot analysis (j) and quantification (k) of cPLA2 and p21 expression in primary human OA chondrocytes treated with various concentrations of VP-16 for 24 hours (n = 3). d,e,g, Data are mean±s.d., *P* values by two-tailed unpaired Student’s *t*-test. i,k, Data are mean±s.d., *P* values by one way ANOVA with Bonferroni post hoc test.


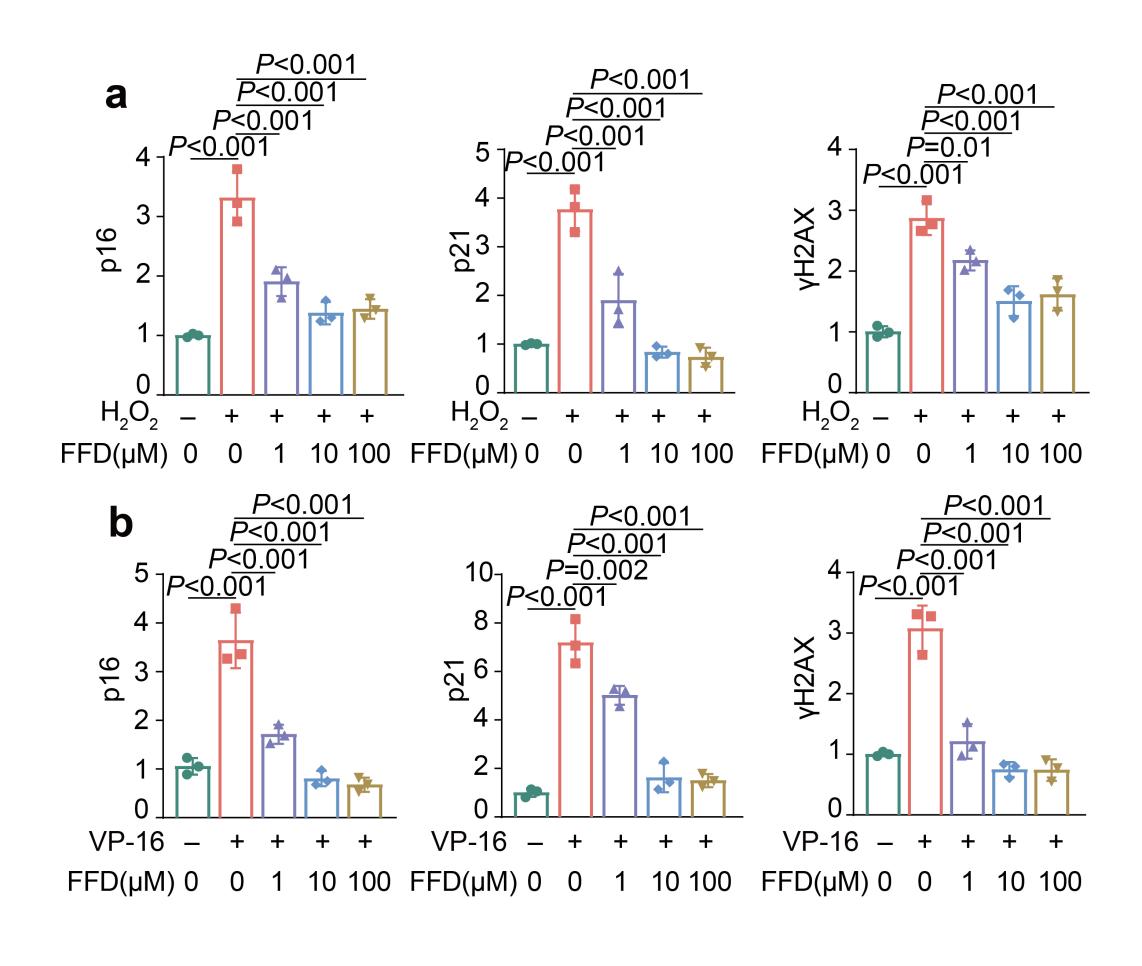


**Fig. S2. Pharmacologically inhibition of cPLA2 regulates the expression of genes related to chondrocyte senescence.** (a) Quantification of p16, p21 and γH2AX and expression in primary human OA chondrocytes treated with 10µM H_2_O_2_ and different concentrations of FFD (n = 3). (b) Quantification of p16, p21 and γH2AX expression in primary human OA chondrocytes treated with 1 ng/mL VP-16 and carying concentrations of FFD (n = 3). a,b, Data are mean±s.d., *P* values by one way ANOVA with Bonferroni post hoc test.


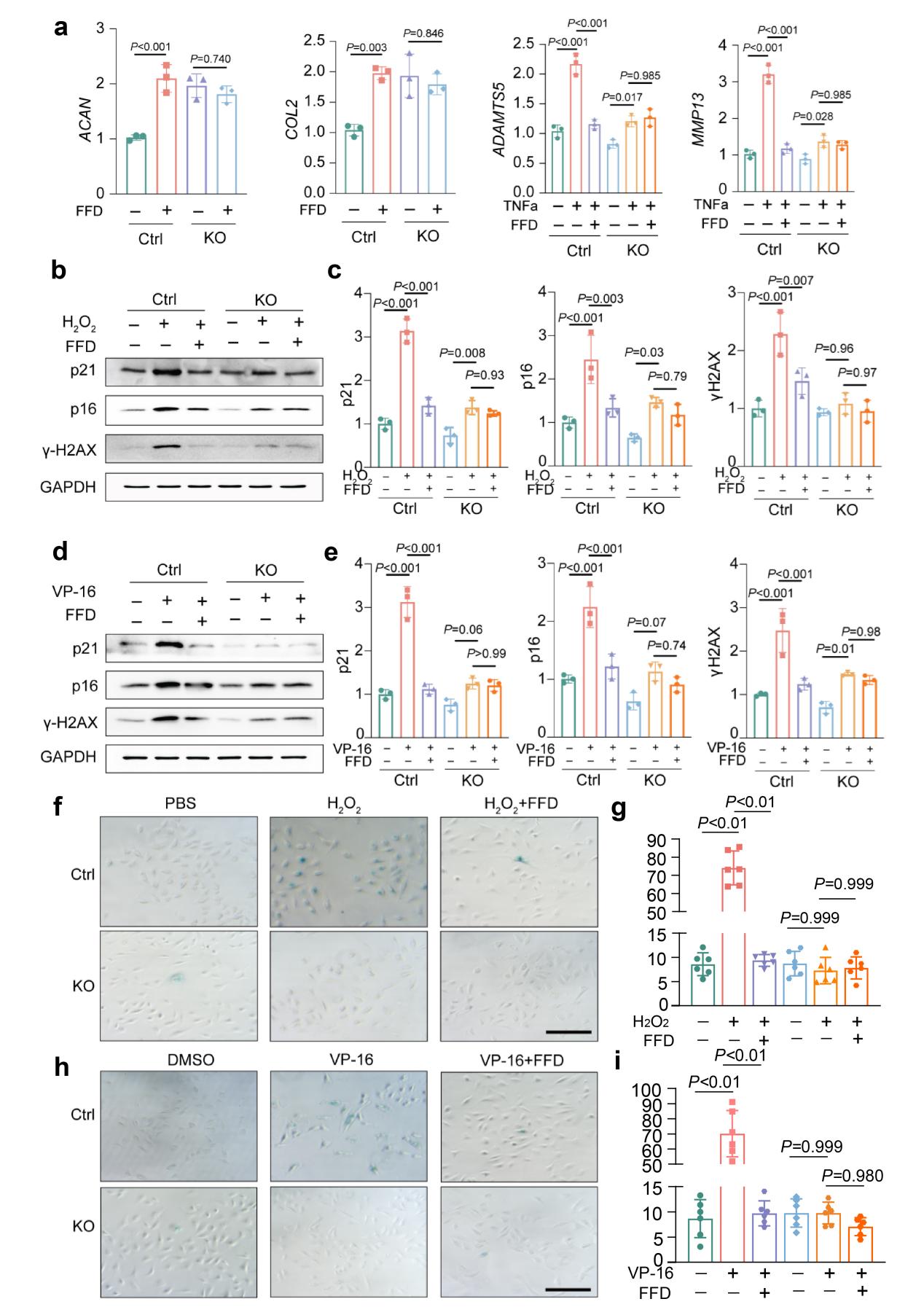


**Fig. S3. FFD regulates chondrocyte metabolism and senescence depending on cPLA2 *in vitro*.** (a) RT-qPCR analysis of *COL2* and *ACAN*, mRNA levels in WT and cPLA2 KO C28/I2 cells treated with or without 10 μM FFD for 24 hrs. mRNA levels of *MMP13* and *ADAMTS5* in WT and cPLA2 KO C28/I2 cells treated with/without 10 ng/ml TNFa in the absence and presence of 10 μM FFD for 24 hrs (n = 3). (b, c) Western blot analysis (b) and quantification (c) of p21, γH2AX, and p16 expression in WT and cPLA2 KO C28/I2 cells treated with/without 10µM H_2_O_2_, and/or 10 μM FFD (n = 3). (d, e) Western blot analysis (d) and quantification (e) of p21, γH2AX, and p16 expression in WT and cPLA2 KO C28/I2 cells treated with/without 1 ng/mL VP-16, and/or 10 μM FFD (n = 3). (f, g) SA-β-gal staining (f) and quantification of SA-β-gal positive cells (g) of WT and cPLA2 KO C28/I2 cells treated with/without 10µM H_2_O_2_ and/or 10 μM FFD for 48h (n = 3). Scale bar = 100 μm. (h-i) SA-β-gal staining (h) and quantification of SA-β-gal positive cells (i) of WT and cPLA2 KO C28/I2 cells treated with 1 ng/mL VP-16 and/or 10 μM FFD for 48h (n = 3). Scale bar = 100 μm. a, c, e, g, i, Data are mean±s.d., *P* values by one way ANOVA with Bonferroni post hoc test.


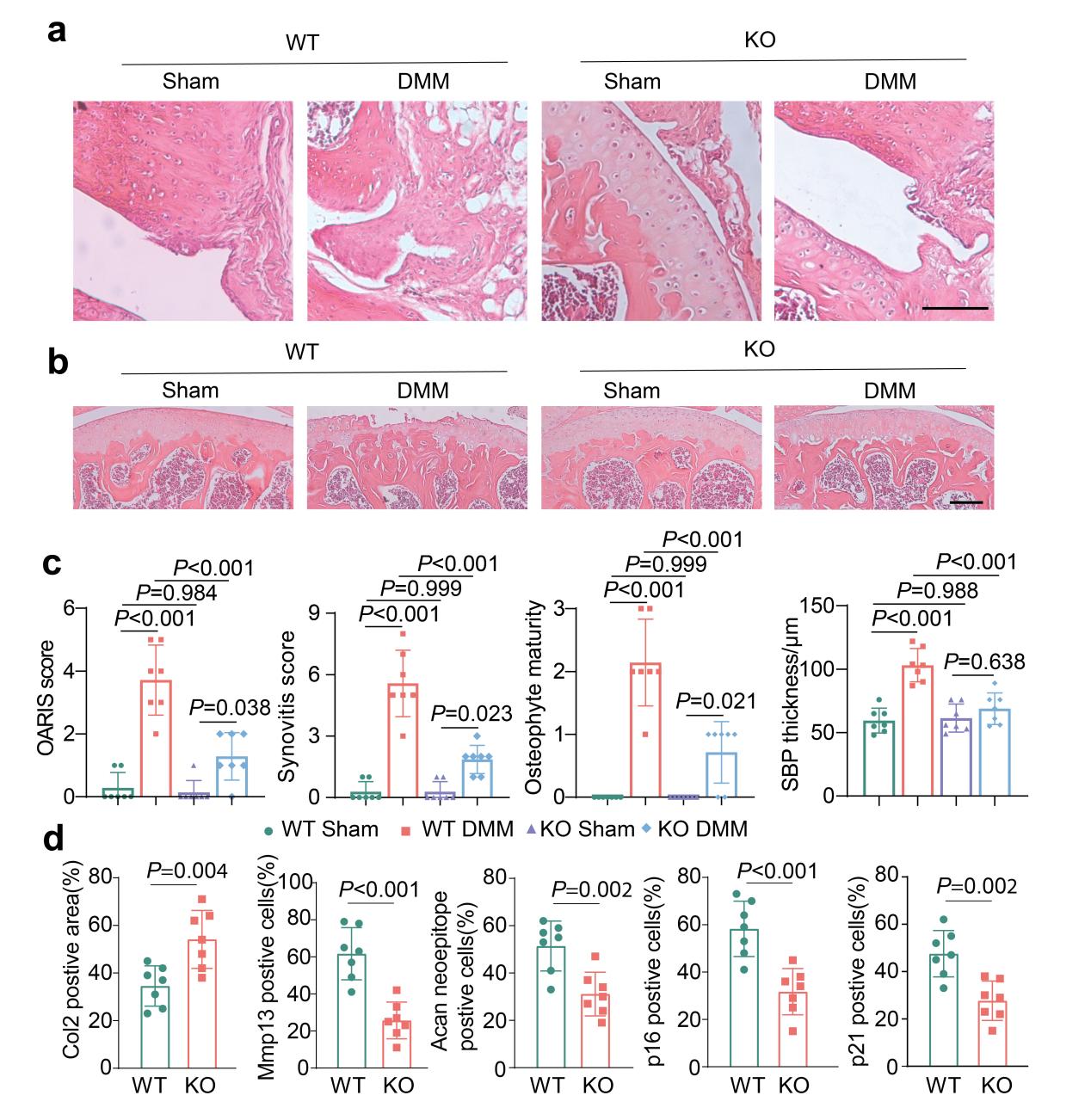


**Fig. S4. Deletion of cPLA2 protects against surgically-induced OA.** (a,b) H&E-stained knee joint sections from WT and cPLA2 KO mice, illustrating the synovium (b) and subchondral bone plate (c) with and without DMM surgery. Scale bar = 100 μm. (c) Quantification of OARSI scores, SBP thickness, osteophyte formation, and synovitis scores in knee joints from WT and cPLA2 KO mice (n = 7). (d) Quantification of positive staining of Col2, MMP13, aggrecan neoepitope, p16, and p21 in knee joint sections from WT and cPLA2 KO mice at 12 weeks after DMM surgery (n = 7). c, Data are mean±s.d., *P* values by one way ANOVA with Bonferroni post hoc test. d, Data are mean±s.d., *P* values by two-tailed unpaired Student’s *t*-test.


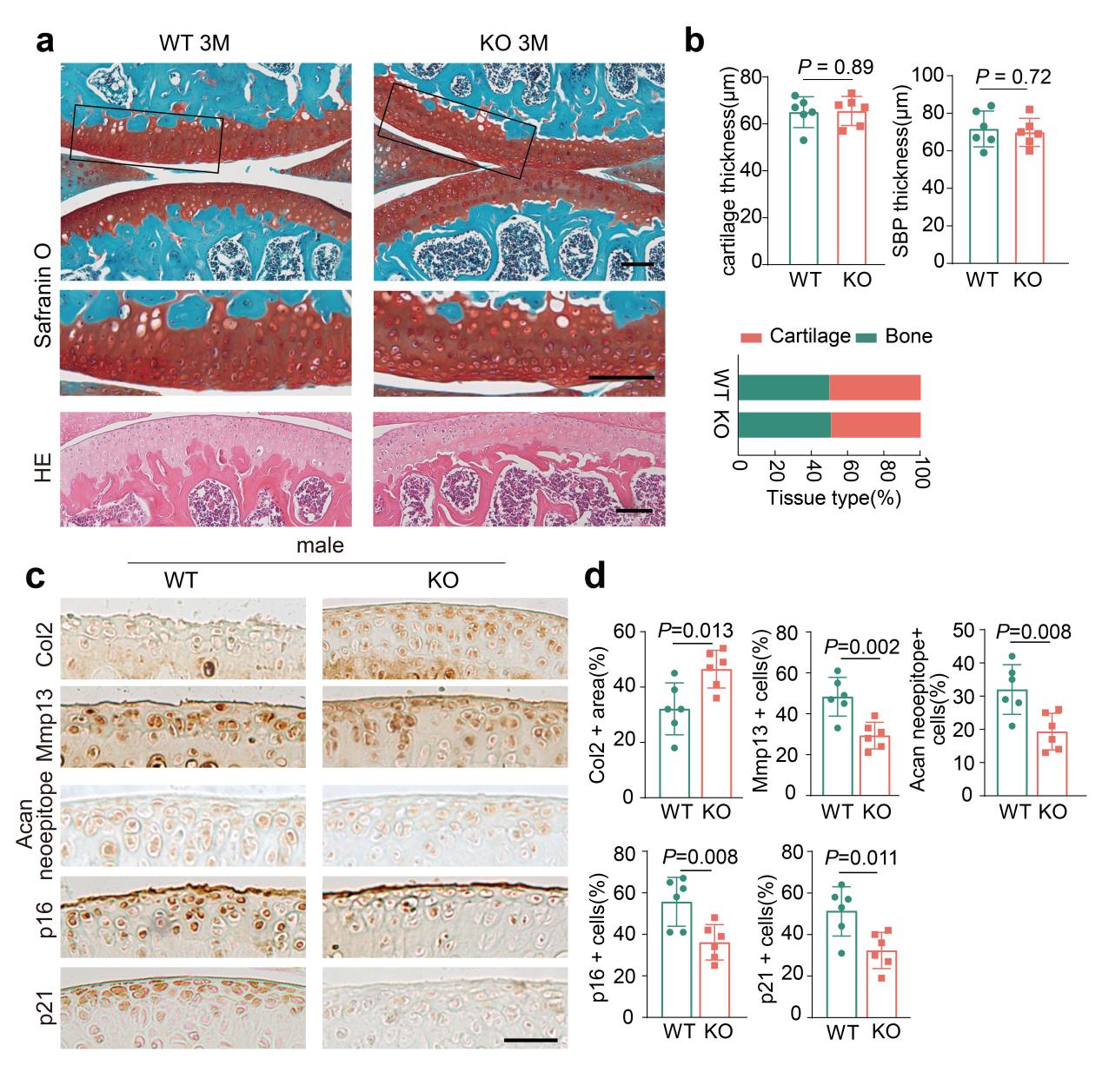


**Fig. S5.** **cPLA2 deficiency does not alter joint morphology in adult mice.** (a) Representative Safranin O/Fast Green and H&E-stained images of knee joints from 3-month-old WT and cPLA2 KO male mice (n = 6). Scale bar = 100 μm. (b) Quantification of cartilage thickness, SBP thickness and tissue type in knee joints from 3-month-old WT and cPLA2 KO male mice (n = 6). **(c,d)** Representative immunohistochemical staining images (c) and quantification (d) of Col2, MMP13, aggrecan neoepitope, p16, and p21 in knee joint sections from 18-month-old WT and cPLA2 KO mice (n = 6). Scale bar = 100 μm. b, d, Data are mean±s.d., *P* values by two-tailed unpaired Student’s *t*-test.

**
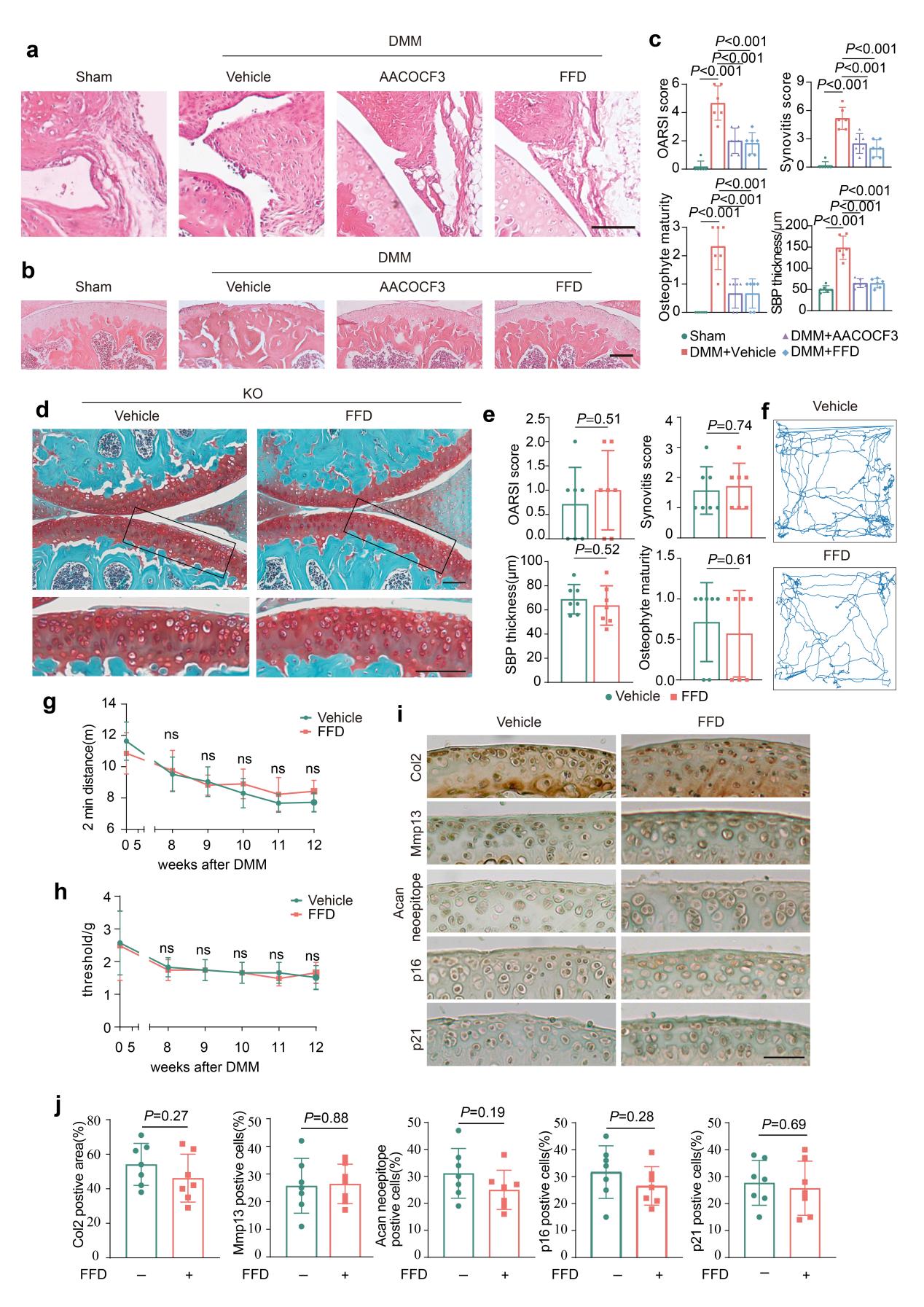
**

**Fig. S6.** **cPLA2 inhibition protects against OA in a surgically induced OA model.** (a-b) H&E-stained knee joint sections from mice treated with the cPLA2 inhibitor AACOCF3 or FFD, depicting synovial tissue (a) and the subchondral bone plate (b). Scale bar = 100 μm. (c) Quantification of OARSI scores, SBP thickness, osteophyte formation, and synovitis scores in knee joints from each group (n = 6). (d) Representative Safranin O/Fast Green and H&E-stained images of knee joints from cPLA2 KO mice treated or untreated with FFD. Scale bar = 100 μm. (e) Quantification of OARSI scores, SBP thickness, osteophyte formation, and synovitis scores in knee joints from cPLA2 KO mice treated or untreated with FFD (n = 7). (f-h) Representative trajectory plots and quantification of 2-minute distance traveled during the open field test (f, g), and quantification of Von Frey test results (h) (n = 7). (i, j) Representative immunohistochemical staining images (i) and quantification (j) of Col2, MMP13, aggrecan neoepitope,p16, and p21 in knee joint sections from cPLA2 KO mice treated or untreated with FFD (n = 7). Scale bar = 50 μm. c, Data are mean±s.d., *P* values by one way ANOVA with Bonferroni post hoc test. **e**, j, Data are mean±s.d., *P* values by two-tailed unpaired Student’s *t*-test.

**
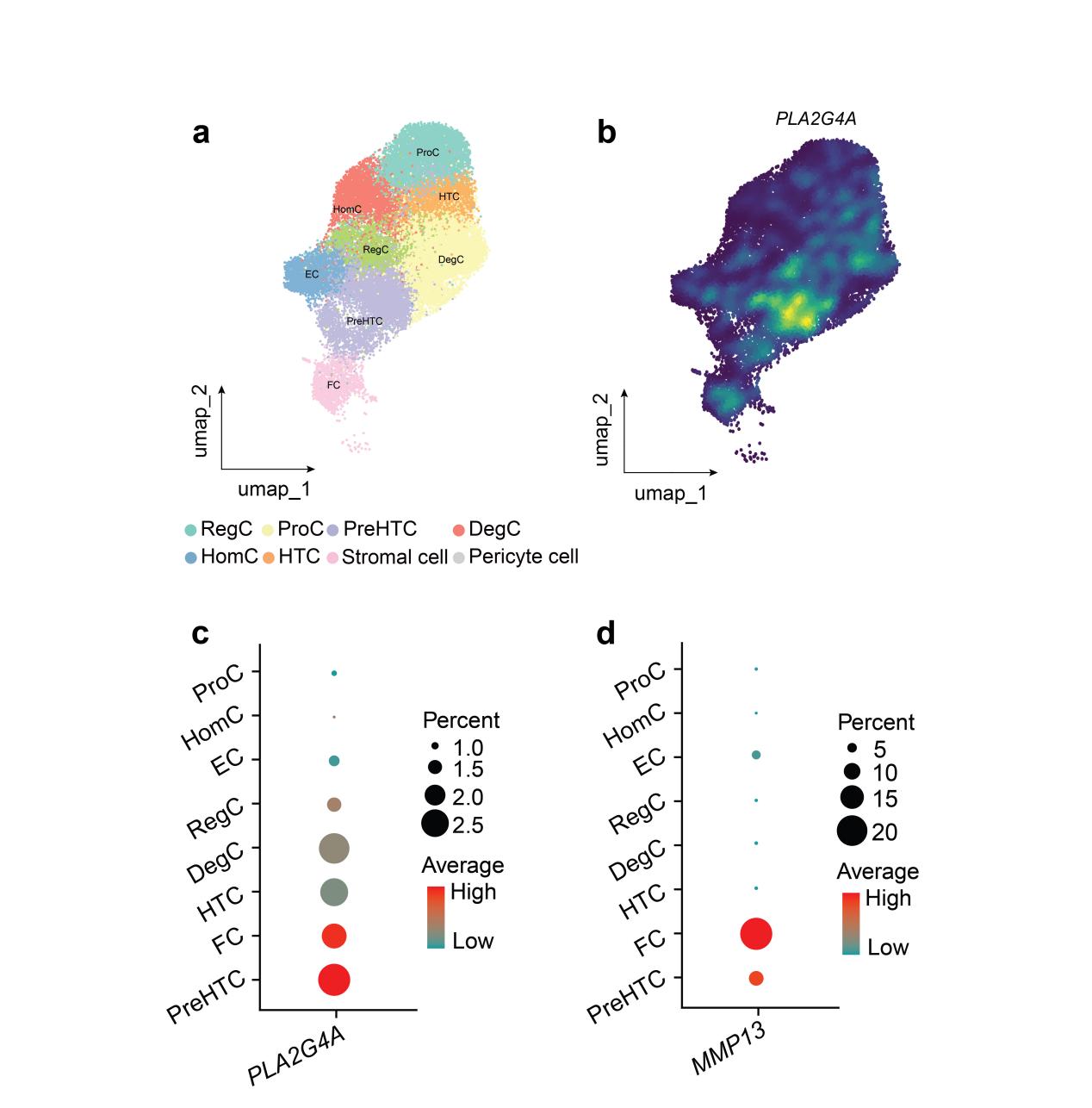
**

**Fig. S7. cPLA2 is mainly expressed in preHTCs of endplate cartilage.** (a) Unbiased clustering of scRNA-seq data from human normal disc endplate revealed 8 distinct cell clusters. (b) Density scatter plot of *PLA2G4A* expression visualized on the UMAP of disc endplate cell. (c-d) Dot plot illustrating the expression patterns of *PLA2G4A* and *MMP13* across diverse chondrocyte subpopulations on endplate cartilage.

**
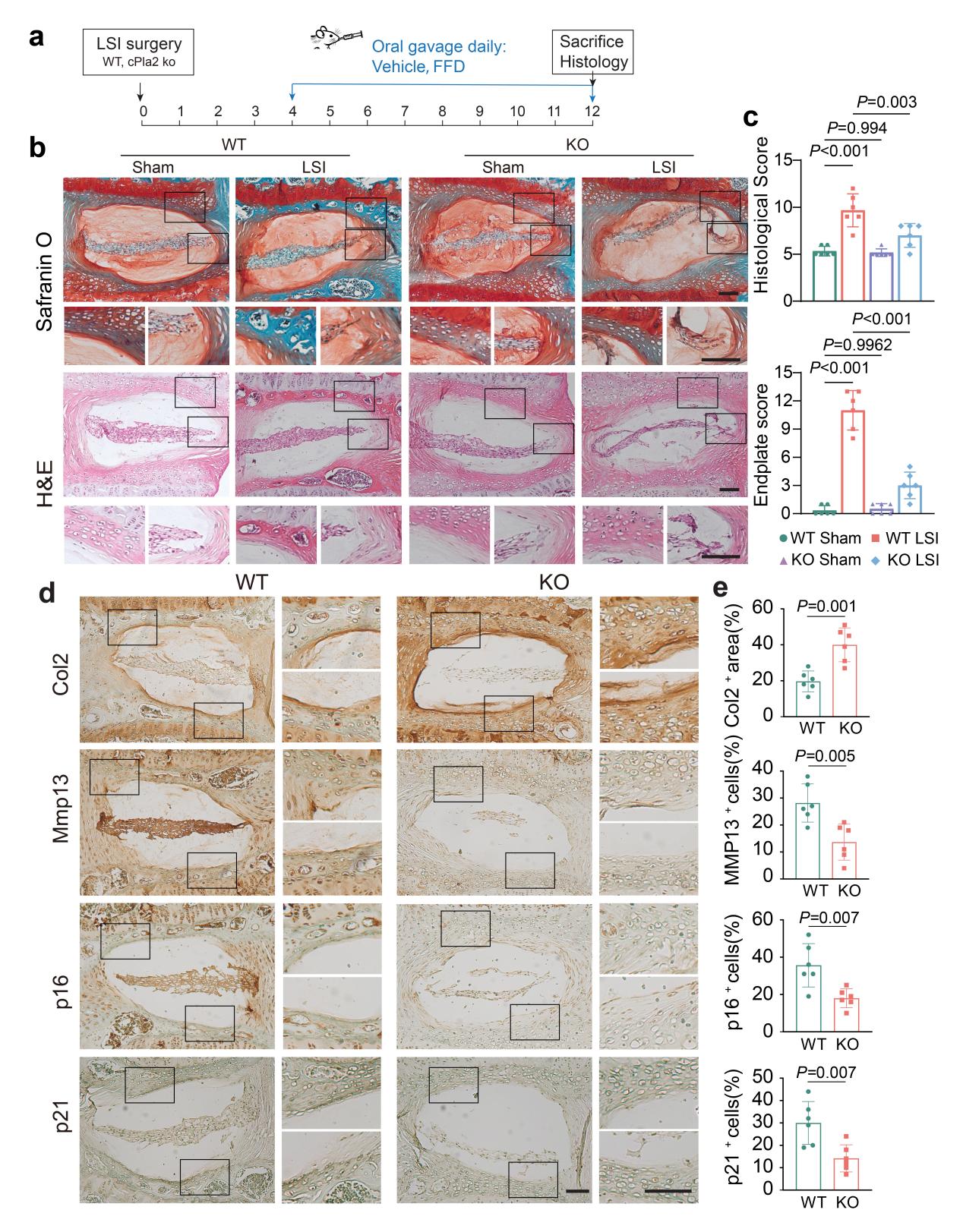
**

**Fig. S8. Genetic deletion of cPLA2 alleviated surgically-induced IVDD.** (a) Schematic of the experimental outline. 12-week-old male WT and cPLA2 KO mice underwent LSI surgery, followed by treatment with/without 10mg/kg body weight FFD daily starting at 4 weeks post-surgery and continuing for 8 weeks (n = 6). (b) Representative Safranin O/Fast Green and H&E-stained images and histological score/Endplate score of intervertebral discs from WT and cPLA2 KO mice (n = 6). Scale bar = 100 μm. (c) Quantification of histological and endplate scores in intervertebral discs from WT and cPLA2 KO mice (n = 6). (d) Representative immunohistochemical staining images and quantification of Col2, Mmp13, p16, and p21 in intervertebral discs sections from each group (n = 6). Scale bar = 100 μm. (e) Quantification of positive staining based on IHC images in d. (n = 6). c, e, Data are mean±s.d., c, *P* values by one way ANOVA with Bonferroni post hoc test. e, *P* values by two-tailed unpaired Student’s *t*-test.


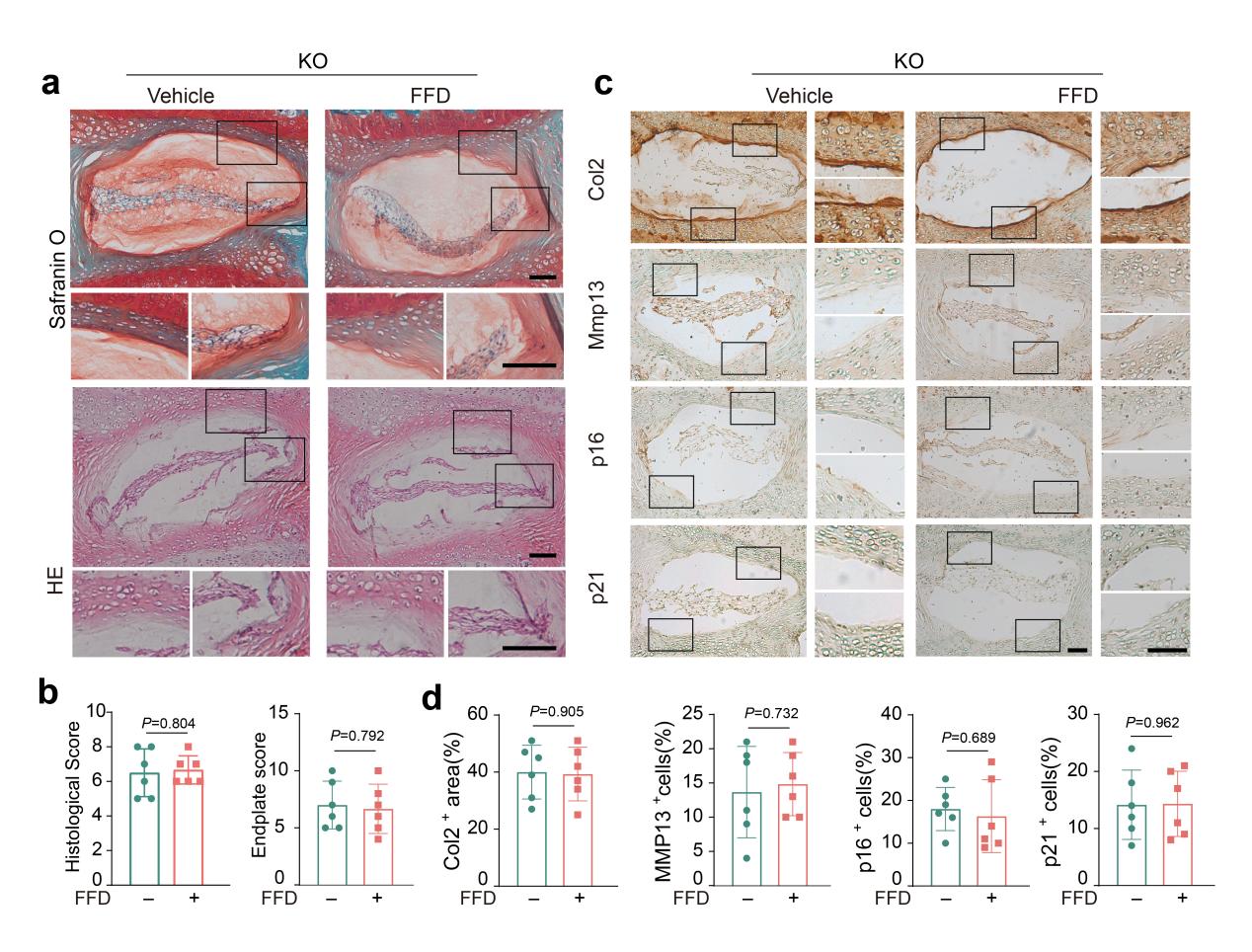


**Fig. S9. FFD protects against IVDD *in vivo* depending on the cPla2 pathway.** (a) Representative Safranin O/Fast Green and H&E-stained images and histological score/Endplate score of intervertebral discs from cPLA2 KO mice treated or untreated with FFD (n = 6). Scale bar = 100 μm. Bottom panel is the magnified view of the highlighted area on the left. (b) Quantification of histological and endplate scores in intervertebral discs from cPLA2 KO mice treated or untreated with FFD (n = 6). (c) Representative immunohistochemical staining images and quantification of Col2, MMP13, p16, and p21 in IVD intervertebral discsfrom each group (n = 6). Right panel is the magnified view of the highlighted area on the left. Scale bar = 100 μm. (d) Quantification of positive staining based on IHC images in c. (n = 6). b, d, Data are mean±s.d., *P* values by two-tailed unpaired Student’s *t*-test.


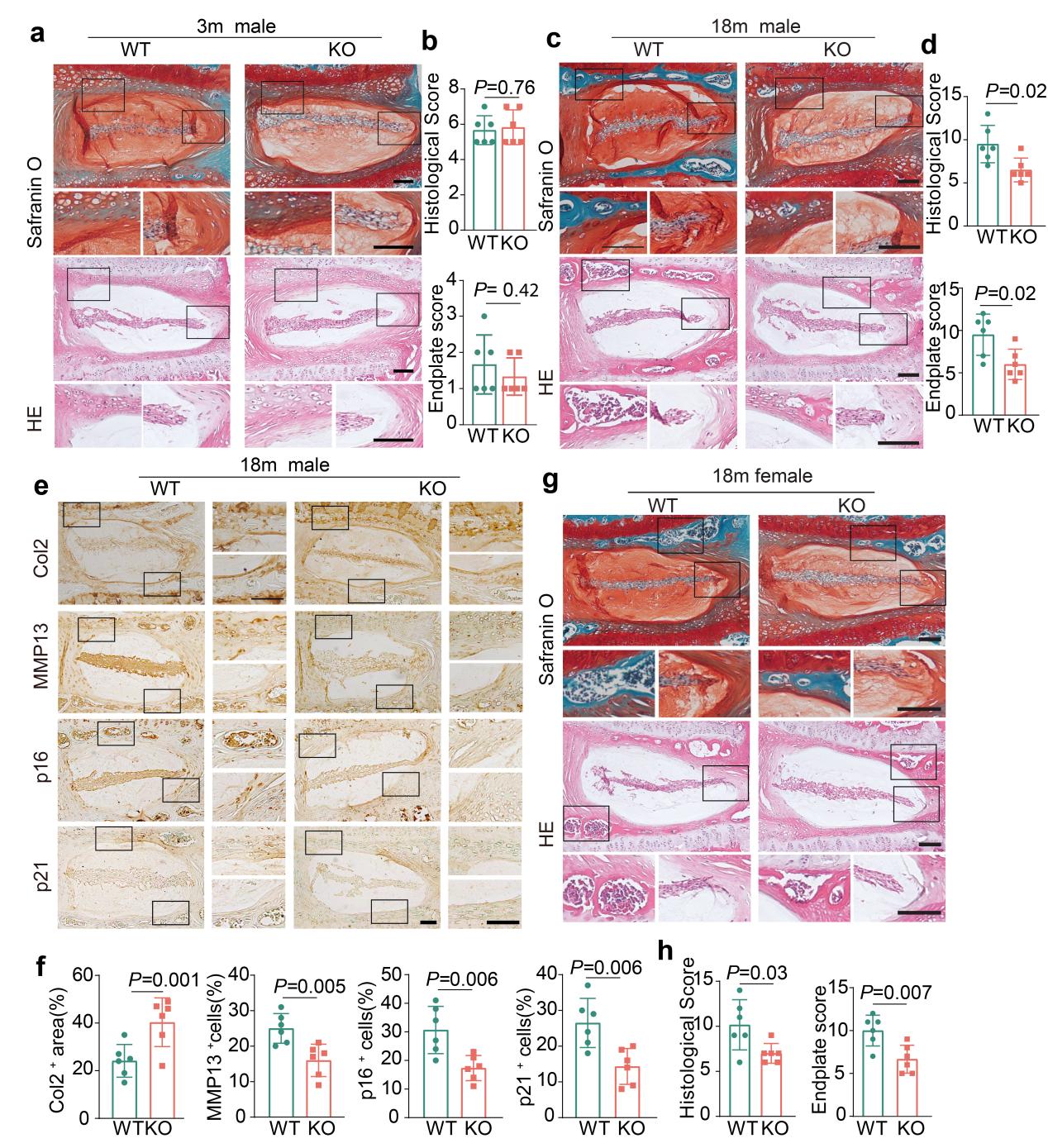


**Fig. S10. Genetic deletion of cPLA2 alleviated age-associated IVDD.** (a) Representative Safranin O/Fast Green and H&E-stained images of intervertebral discs from 3-month-old WT and cPLA2 KO male mice (n = 6). Scale bar = 100 μm. (b) Quantification of histological and endplate scores in intervertebral discs from 3-month-old WT and cPLA2 KO male mice (n = 6). (c) Representative Safranin O/Fast Green and H&E-stained images of intervertebral discs from 18-month-old WT and cPLA2 KO male mice (n = 6). Scale bar = 100 μm. (d) Quantification of histological and endplate scores in intervertebral discs from 18-month-old WT and cPLA2 KO male mice (n = 6). (e) Representative immunohistochemical staining images of Col2, MMP13, p16, and p21 from 18-month-old WT and cPLA2 KO male mice (n = 6). Scale bar = 100 μm. (f) Quantification of positive staining of COl2, MMP13, p16, and p21 in knee joint sections in e (n = 6). (g) Representative Safranin O/Fast Green and H&E-stained images of intervertebral discs from 18-month-old WT and cPLA2 KO female mice (n = 6). Scale bar = 100 μm. (h) Quantification of histological and endplate scores in intervertebral discs from 18-month-old WT and cPLA2 KO female mice (n = 6). b,d,f,h, Data are mean±s.d., *P* values by two-tailed unpaired Student’s *t*-test.

**Supplemental Table 1.** OA- and senescence-associated secretory phenotype (SASP)-related gene sets (1)

| OA-related gene sets | senescence-associated secretory phenotype (SASP)-related gene sets |
| --- | --- |
| AREG, EREG, NRG1, HGF, TNFRSF1A, SERPINE2, ANG, CCL4, SERPINE1, TIMP2, PLAT, PNG, MIF, MMP14, MMP13, MMP12, IL6, IL1B, IL18, IL15, IGFBP7, IGFBP6, IGFBP5, IGFBP4, IGFBP3, IGFBP2, ICAM1, HMGB1, CXCL16, CXCL12, CXCL2, CXCL1, CTNNB1, CCL5, CCL3, CCL2 | HLADRA, ITGB2, NOV, TUBB3, GPR68, CYR61, TGFBI, CDH11, COL2A1, ANLN, ANPEP, PART1, MEX3D, PREX2, PENK, NPR3, MKI67, RSPO2, OGN, LRRC15, P4HA3, COL8A1, NPTX2, MMP13, ITGB8, MXRA5, COL1A2, FAP, KIAA1644, TNR, TMEM59L, ARHGAP9, SMOC2, SHC4, DIO2, CDH10, FAT3, CACNA1A, ST6GAL2, NOTCH3, TTC9, PDGFC, RRM2, MYO3A, SFRP4, ECT2, SEMA3C, CDH2, HTRA1, C4BPA, AMTN, FLRT2, ADAMTS7, TREM1, POSTN, THY1, CSN1S1, MMP11, LOC441666, SNX10, KCNN4, FRMD7, COL1A1, GJB2, RUNX1IT1, SYNDIG1, ADAMTS14, TPPP3, PRSS23, THBS2, CEP55, TOP2A, KCNS3, IGFBP3, CRLF1, GFRA2, TYMP, BUB1, WNT5A, NELL1, HMGA2, NCAM1, ST6GALNAC5, SULF1, BASP1, MMP19, INSC, TLL1, TNC, ISM1AS1, SPOCK1, CDK1, CLEC3B, THEMIS2, COL3A1, GRIA2, FNDC1, CFI, MME, TMEM119, PPP1R14C, NAT2, PAQR4, CCND1, SPP1, DOCK10, KIF20A, SLC9C1, SERPINE2, TNFSF15, SEZ6L2, CENPF, ADAMTS2, CTHRC1, LOC400940, EFEMP1, KIF5C, MATN3, SOX11, CHI3L2, S100A4, TNFAIP6, TNFSF11, RARRES1, DIAPH3, ASPM, DMBT1, EPDR1, IGFBP1, GRIN2A, RAMP3, OLFML2B, ENC1, CTSC |

**Supplemental Table 2. Primer sequences used for quantitative real-time PCR**

| Gene | Forward primer | Reverse primer |
| --- | --- | --- |
| *GAPDH* | ACGGGAAGCTTGTCATCAAT | TGGACTCCTCGACGTACTCA |
| *cPLA2* | CCTTTCTCTGGAAAATCAGGGTG | GGATTCTCTGGTGTGATGAAGGC |
| *ACAN* | GGGAAGGCTGCTATGGAGAC | ACCTCACCCTCCATCTCCTC |
| *COL2A1* | CTGGAAAAGCTGGTGAAAGG | GGCCTGGATAACCTCTGTGA |
| *MMP13* | TGACCCTTCCTTATCCCTTG | ATACGGTTGGGAAGTTCTGG |
| *ADAMTS5* | TTTGGACCAGGGCTTAGATG | CTTCACTGTGGCTCACGAAA |

Abbreviations: GAPDH, glyceraldehyde-3-phosphate dehydrogenase; *cPLA2*, Cytosolic phospholipase A2; ACAN, aggrecan; COL2A1, collagen type II alpha 1 chain; ADAMTS5, ADAM metallopeptidase with thrombospondin type 1 motif 5; MMP13, matrix metallopeptidase 13.

**Reference**

1. Swahn H, Li K, Duffy T, Olmer M, D'Lima DD, Mondala TS, et al. Senescent cell population with ZEB1 transcription factor as its main regulator promotes osteoarthritis in cartilage and meniscus. Ann Rheum Dis. 2023;82(3):403-15.
